# Neuronal activity rapidly reprograms dendritic translation via eIF4G2:uORF binding

**DOI:** 10.1038/s41593-024-01615-5

**Published:** 2024-04-08

**Authors:** Ezgi Hacisuleyman, Caryn R. Hale, Natalie Noble, Ji-dung Luo, John J. Fak, Misa Saito, Jin Chen, Jonathan S. Weissman, Robert B. Darnell

**Affiliations:** 1 Laboratory of Molecular Neuro-oncology, The Rockefeller University, New York, NY USA; 2 Bioinformatics Resource Center, The Rockefeller University, New York, NY USA; 3 Department of Pharmacology and Cecil H. and Ida Green Center for Reproductive Biology Sciences, The University of Texas Southwestern Medical Center, Dallas, TX USA; 4 Whitehead Institute for Biomedical Research, Cambridge, MA USA; 5 Department of Biology, Massachusetts Institute of Technology, Cambridge, MA USA; 6 Howard Hughes Medical Institute, Massachusetts Institute of Technology, Cambridge, MA USA; 7 David H. Koch Institute for Integrative Cancer Research, Massachusetts Institute of Technology, Cambridge, MA USA; 8 Howard Hughes Medical Institute, The Rockefeller University, New York, NY USA; 9 Present Address: Memorial Sloan Kettering Cancer Center, New York, NY USA; 10 Present Address: Altos Labs, Bay Area Institute of Science, Redwood City, CA USA

**Keywords:** Molecular neuroscience, Ribosome

## Abstract

Learning and memory require activity-induced changes in dendritic translation, but which mRNAs are involved and how they are regulated are unclear. In this study, to monitor how depolarization impacts local dendritic biology, we employed a dendritically targeted proximity labeling approach followed by crosslinking immunoprecipitation, ribosome profiling and mass spectrometry. Depolarization of primary cortical neurons with KCl or the glutamate agonist DHPG caused rapid reprogramming of dendritic protein expression, where changes in dendritic mRNAs and proteins are weakly correlated. For a subset of pre-localized messages, depolarization increased the translation of upstream open reading frames (uORFs) and their downstream coding sequences, enabling localized production of proteins involved in long-term potentiation, cell signaling and energy metabolism. This activity-dependent translation was accompanied by the phosphorylation and recruitment of the non-canonical translation initiation factor eIF4G2, and the translated uORFs were sufficient to confer depolarization-induced, eIF4G2-dependent translational control. These studies uncovered an unanticipated mechanism by which activity-dependent uORF translational control by eIF4G2 couples activity to local dendritic remodeling.

## Abstract (web summary)

Precise profiling of dendritic RNA regulation reveals how neuronal depolarization leads to ribosome switching onto short upstream open reading frames and new coding sequences to acutely modulate local protein synthesis.

## Main

The complex and highly elongated structure of neurons renders subcellular regions subject to local demands. mRNA localization in neurites is thought to play a critical role in neuronal homeostasis and synaptic plasticity^1,2^, and activity-dependent changes in translation are required to drive synaptic plasticity, learning and memory^3–6^. However, with current molecular tools, the dynamics of synaptic metabolism and molecular plasticity are not fully understood.

Imaging approaches established the groundwork for the discovery of mRNAs in neurites^7,8^. The subcellular localization and translation of these RNAs have been studied using mechanical separation methods^9,10^. Enzymatic tagging has recently enabled the cell-type-specific analysis of protein composition of subcellular compartments^11–13^. Studies in resting neurons revealed the unique biology of localized transcripts, their isoform specificity and 5′ and 3′ untranslated region (UTR) regulation by RNA-binding proteins (RBPs). Dendritically localized RNAs, for example, have longer 5′ UTRs^14^, which can enable complex translational programs by forming structural motifs and interacting with RBPs. Association of these RBPs with 5′ UTRs correlates with the translation dynamics of neuronal transcripts^15^. Sequence elements within the 5′ UTRs impact ribosome engagement^16,17^ and translation of the downstream coding sequence (CDS) in response to different stimuli and cellular states^18–20^. However, it is not known if and how 5′ UTRs or other mechanisms modulate CDS translation of dendritically localized messages.

A longstanding goal in neuroscience is to understand how the localization of dendritic RNAs leads to protein synthesis-dependent synaptic plasticity. In the present study, we developed proximity-based labeling methods to simultaneously isolate dendritic RNAs and their bound regulatory proteins, along with dendritic ribosomes and proteins, to investigate how neuronal depolarization impacts molecular events in synapses. We localized a biotin ligase to postsynapses and analyzed local molecular changes within 20–30 min after depolarization with either glutamate receptor agonist DHPG or KCl. Surprisingly, depolarization induced the translation of many short upstream open reading frames (uORFs) and downstream CDSs in transcripts that were dormant in resting dendrites. Motif analysis and crosslinking immunoprecipitation (CLIP) revealed that the 5′ UTRs harboring these uORFs were bound by eIF4G2, and mutation studies uncovered that its binding was necessary and sufficient for the downstream CDS translation. Taken together, with these molecular tools, we were able to reveal previously unanticipated aspects of localized activity-dependent translational control with spatial and temporal resolution.

## Results

### Dendritic TurboID identifies postsynaptic RNAs and proteins

**Fig. 1:**
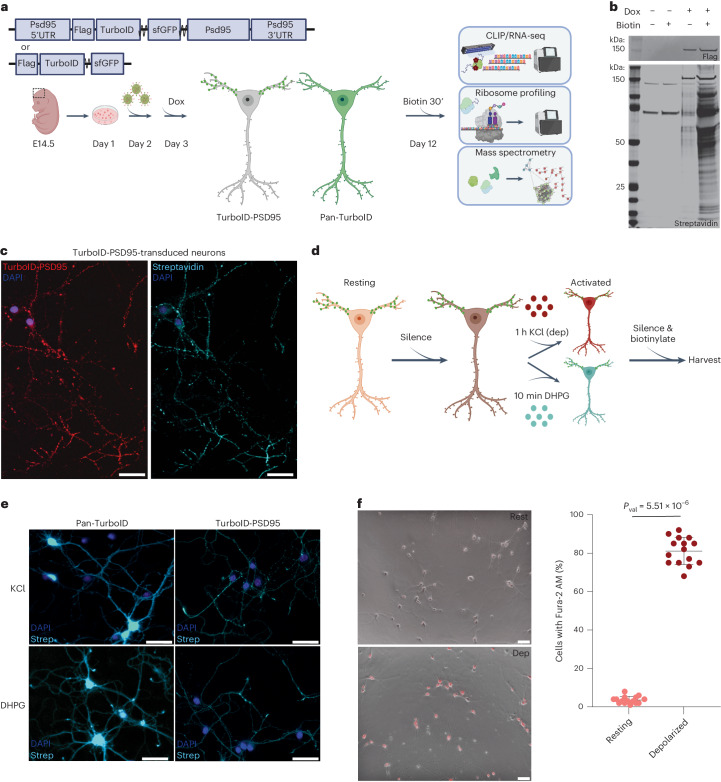
TurboID is a robust methodology to isolate the molecular composition of the postsynaptic compartment in primary cortical neurons. **a**, The implementation of TurboID in primary cortical neurons. **b**, Doxycycline (Dox) induction of TurboID-PSD95 (Flag) and its biotinylation pattern (streptavidin) in the presence and absence of biotin shown by western blots. **c**, IF to detect TurboID expression and biotinylation in primary cortical neurons transduced with TurboID-PSD95 after 30 min of biotin incubation. DAPI (blue) marker for nuclei, Flag (red) stain for TurboID and streptavidin (cyan) to demarcate the biotinylated proteins. Magnification, ×40. **d**, Diagram of neuronal activation workflow with TurboID labeling: neurons are first silenced with TTX (sodium channel blocker) and DL-AP5 (NMDA receptor antagonist) to standardize activity levels in culture; then, activated with KCl (dep) for 1 h or by DGPH for 10 min; and, finally, incubated with the same silencers and biotin to induce biotinylation and allow for recovery (30 min for KCl and 20 min for DHPG). Colors: salmon (resting); burgundy (depolarized by KCl); cyan (DHPG). **e**, Distribution of streptavidin signal (cyan) in Pan-TurboID-transduced or TurboID-PSD95-transduced activated neurons. DAPI (blue) marker for nuclei. Magnification, ×20. **f**, Fura-2 AM staining (left) and quantification (right) in resting and KCl-depolarized neurons. Each circle represents information from one field (data are mean ± s.d., 15 fields total from three biological replicates). Significance was derived from the biological replicates and calculated using the two-tailed, unpaired Student’s *t*-test. Scale bars, 50 μm. Source data

**Extended Data Fig. 1:**
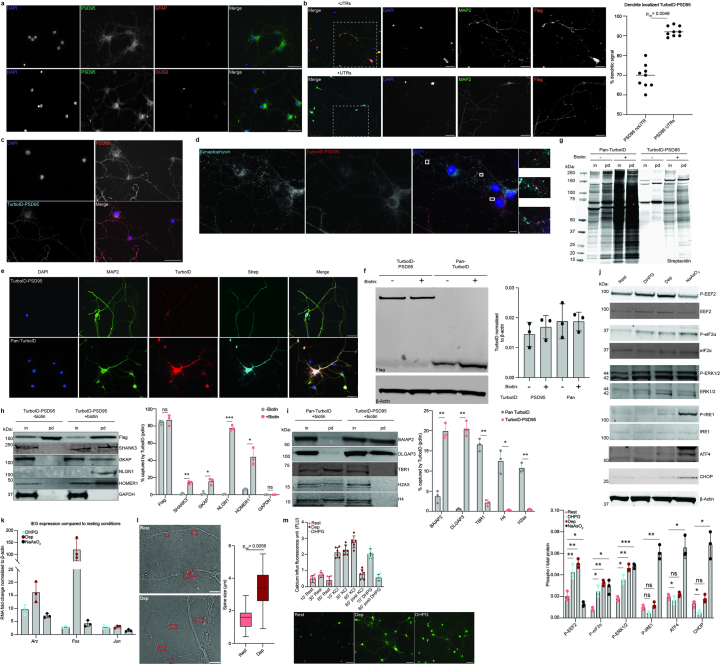
Labeling by TurboID and activation by KCl or DHPG are used to study resting and activated dendritic molecular profiles in primary cortical neurons. **a**, Immunofluorescence (IF) images of primary cortical neurons immunostained for glial fibrillary acidic protein (GFAP) and oligodendrocyte transcription factor 2 (OLIG2) simultaneously with PSD95 to show that the cultures are devoid of glial cells or oligodendrocytes, respectively. DAPI for nuclei; PSD95 for excitatory neurons. Magnification, ×40. Scale bars, 50 μm. **b**, TurboID-PSD95 was cloned without (top row) and with (bottom row) its 5′ and 3′ UTRs and lentivirally expressed in primary cortical neurons. White dashed boxes are zoomed in areas in black&white images. DAPI for nuclei; MAP2 for dendrites; Flag for each TurboID. % dendritically localized TurboID-PSD95 is quantified by co-localization with MAP2 signal in ImageJ. 3 different areas of images per replicate (*n* = 3). Magnification, ×20. Scale bars, 50 μm. Significance was derived from biological replicates, showing the center line at mean. **c**, IF images of TurboID-PSD95-transduced neurons immunostained for DAPI (blue, for nuclei), PSD95 (red, for endogenous PSD95) and TurboID-PSD95 (cyan, detected by Flag). Magnification, ×60. Scale bar, 50 μm. **d**, IF images show the expression of a presynaptic marker, Synaptophysin (cyan), and TurboID-PSD95 (red, detected by Flag antibody) in primary cortical neurons transduced with TurboID-PSD95. DAPI (blue) marker for nuclei. Three zoomed in regions are marked by the white boxes. Magnification, ×60. Scale bar, 10 μm. **e**, IF images show TurboID expression and biotinylation in primary cortical neurons transduced with TurboID-PSD95 or Pan-TurboID after 30 minutes of biotin incubation. DAPI (blue, nuclei); MAP2 (green, dendrites); Flag (red, TurboID); and Streptavidin (cyan, biotinylated proteins). Magnification, ×20. Scale bars, 50 μm. **f**, Western blots stained for Flag and β-Actin from Pan-TurboID and TurboID-PSD95-transduced neurons in the absence (−) or presence (+) of exogenous biotin shown to indicate the relative expression levels of TurboID proteins. Quantifications of TurboID protein levels normalized to β-Actin are shown on the right (*n* = 3); relative levels are not significant by two-tailed, paired Student’s *t*-test. **g**, Western blots stained for streptavidin signal in inputs (‘in’) and streptavidin pulldowns (‘pd’) from Pan-TurboID or TurboID-PSD95-transduced neurons in the absence (−) or presence (+) of exogenous biotin. **h**, Streptavidin pulldowns shown for dendritic (SHANK3, GKAP, NLGN1 and HOMER1) and negative control (GAPDH) proteins from TurboID-PSD95-transduced neurons in the absence (−) or presence (+) of exogenous biotin. Flag signal indicates self-biotinylation of each construct. Loaded on the gel are 10% (by volume) of input and 50% (by volume) of pulldowns. Percent isolated by TurboID-PSD95 in each condition is calculated by dividing the signal in the pulldown lane by that of the input lane, after each is adjusted to total, and quantifications are shown as bar graphs (*n* = 3). *P* values: Flag = 0.58, SHANK3 = 0.0061, GKAP = 0.018, NLGN1 = 0.00052, HOMER1 = 0.021, GAPDH = 0.42. **i**, Streptavidin pulldowns shown for dendritic (BAIAP2 and DLGAP3) and nuclear (TBR1, H4 and H2AX) proteins from Pan-TurboID and TurboID-PSD95-transduced neurons in the presence (+) of exogenous biotin. Loaded on the gel are 10% (by volume) of input and 50% (by volume) of pulldowns. Percent isolated by each TurboID is calculated as in **(h)** (*n* = 3). *P* values: BAIAP2 = 0.0052, DLGAP3 = 0.0035, TBR1 = 0.0063, H4 = 0.018, H2AX = 0.0037. **j**, Phosphorylation of EEF2, eIF2α, ERK1/2 and IRE1 and total levels of ATF4 and CHOP are shown in resting (rest), activated (DHPG, Dep) and stressed (Sodium arsenite (NaAsO_2_)) cells by using phospho-specific and total antibodies. The amount of phosphorylated or total protein is shown in the bar graphs, calculated by dividing the phosphorylated signal to total and β-Actin for the phosphorylated proteins and by dividing the total to β-Actin for ATF4 and CHOP (*n* = 3). Significance was calculated with respect to rest. *P* values: P-EEF2 (DHPG = 0.0088, Dep = 0.0023, NaAsO_2_ = 0.039), P-eIF2α (DHPG = 0.018, Dep = 0.0034, NaAsO_2_ = 0.028), P-ERK1/2 (DHPG = 0.015, Dep = 0.0067, NaAsO_2_ = 0.00084), P-IRE1 (DHPG = 0.06, Dep = 0.37, NaAsO_2_ = 0.0027), ATF4 (DHPG = 0.038, Dep = 0.42, NaAsO_2_ = 0.016), CHOP (DHPG = 0.044, Dep = 0.18, NaAsO_2_ = 0.024). **k**, Quantitative PCR (qPCR) results shown for immediate early genes, *Arc*, *Fos* and *Jun*. The fold changes for each gene are calculated by first normalizing to the house-keeping gene *β-Actin* in each condition, then dividing the value of each condition by that of the resting state (*n* = 3). **l**, Dendritic spine size in resting and KCl-depolarized neurons are measured using the Keyence microscope. Red squares are examples of spines that are counted (*n* = 3, 12 spines from each biological replicate are counted as technical replicates). Significance was derived from the biological replicates using the two-tailed, unpaired Student’s *t*-test. Box plots show the min and max, with the center line at median. Magnification, ×100. Scale bars, 5 μm. **m**, Fluo-4-AM staining in resting, KCl-depolarized and DHPG-depolarized cells. Fluo4-AM was loaded in resting cells and measurements were taken at indicated time points after Fluo4-AM removal. In depolarized cells, the dye was loaded during silencing. After silencing, fluorescence was measured during stimulus at 10, 30 and 60-minute time points for the KCl treatment and at 10-minute for the DHPG-induced activation. Fluorescence was also measured 60 minutes after the stimulus removal (60′post KCl and 60′post DHPG). Circles represent data from 2 biological and 3 technical replicates. Below: Examples of Fluo4-AM fluorescence are shown in resting, 10-minute KCl-treated and 10-minute DHPG-treated neurons. Fluo4-AM loading (45 minutes) was performed during the last 45 minutes of the silencing step prior to stimulus addition for the KCl and DHPG treatment and simultaneously for the resting neurons. Imaging was performed 10 minutes after the stimulus was added. Scale bars, 50 μm. **(b,f,h-k,m)** Data are mean ± s.d. Significance was calculated using the two-tailed, paired Student’s *t*-test. *P* values: ns (not significant) >0.05; * <0.05; ** <0.01; *** <0.001; **** <0.0001. *n* indicates the number of biologically independent samples. Source data

We used a bio-orthogonal and kinetically enhanced biotin ligase, TurboID^21^, to specifically biotinylate proteins within a 10–50 nm radius. We engineered a tagged TurboID to confer dendritic localization by linking it to the elements of the transcript encoding the postsynaptic protein PSD95 (TurboID-PSD95) (Fig. 1a), expressed from a doxycycline-inducible lentiviral construct. First, we confirmed that our culture was devoid of contaminating non-neuronal cell types (Extended Data Fig. 1a). The palmitoylation signal and UTRs of PSD95 were necessary for the proper and punctate dendritic localization of TurboID-PSD95, consistent with previous studies^22^ (Extended Data Fig. 1b–d). The qualitative and quantitative immunofluorescence (IF) data confirmed that TurboID-PSD95 is localized to dendritic puncta despite some variation in the intensity of expression across neurons. In resting neurons, exogenous addition of biotin induced robust biotinylation and local labeling of nearby proteins in 30 min, as visualized by streptavidin pulldowns and IF (Fig. 1b,c and Extended Data Fig. 1e).

To demonstrate the localization and specificity of TurboID-PSD95, we compared it to TurboID without a localization signal (Pan-TurboID; Extended Data Fig. 1e,f), which is similarly expressed and records information from the whole neuron. After addition of biotin, both Pan-TurboID and TurboID-PSD95 efficiently labeled nearby proteins, albeit with more background labeling with Pan-TurboID even in the absence of additional biotin (Fig. 1c and Extended Data Fig. 1e,g). Streptavidin pulldowns followed by western blots showed that TurboID-PSD95 specifically biotinylated and allowed the recovery of known dendritic binding partners of PSD95 but not non-localized proteins (Extended Data Fig. 1h)^23^. Comparison of the enrichment of dendrite-localized and nuclear proteins isolated by TurboID-PSD95 revealed up to 30-fold purification of dendritic proteins compared to Pan-TurboID (Extended Data Fig. 1i). Taken together, these data demonstrate that proximity labeling with TurboID-PSD95 was highly efficient in identifying dendritic proteins in resting neurons.

Activity-driven RNA localization and translation in dendrites is crucial for tuning the synaptic proteome and inducing long-lasting modifications of neuronal circuits; however, little is known about the dynamics of postsynaptic RNA regulation after neuronal activation. To bridge this gap, we used KCl^24^ (referred to as ‘dep’) or the specific glutamate agonist DHPG^25^ to acutely depolarize neurons (Fig. 1d,e). We optimized parameters based on the increase in the phosphorylation of known translational control and signal transduction pathway components, transcription of immediate early genes (IEGs) and dendritic spine lengthening while also allowing time for ample TurboID biotinylation (Extended Data Fig. 1j–l). Neuronal depolarization was assessed by monitoring increased intracellular calcium concentration, demonstrating activation of the majority (~80%) of neurons (Fig. 1f).

Depolarization by KCl and DHPG resulted in similar biotinylation, phosphorylation, IEG expression and calcium influx (Fig. 1e and Extended Data Fig. 1j,k,m). We compared these activation paradigms with sodium arsenite, which specifically induces stress responses, and found that depolarization led to distinct changes (IEGs and P-EEF2) that distinguished it from a classical stress response (activation of IRE1, CHOP and ATF4) (Extended Data Fig. 1j,k). Based on these data, our strategy provides a means to measure specific molecular changes in dendrites in response to depolarization and to probe the unusual nature of these responses.

**Extended Data Fig. 2:**
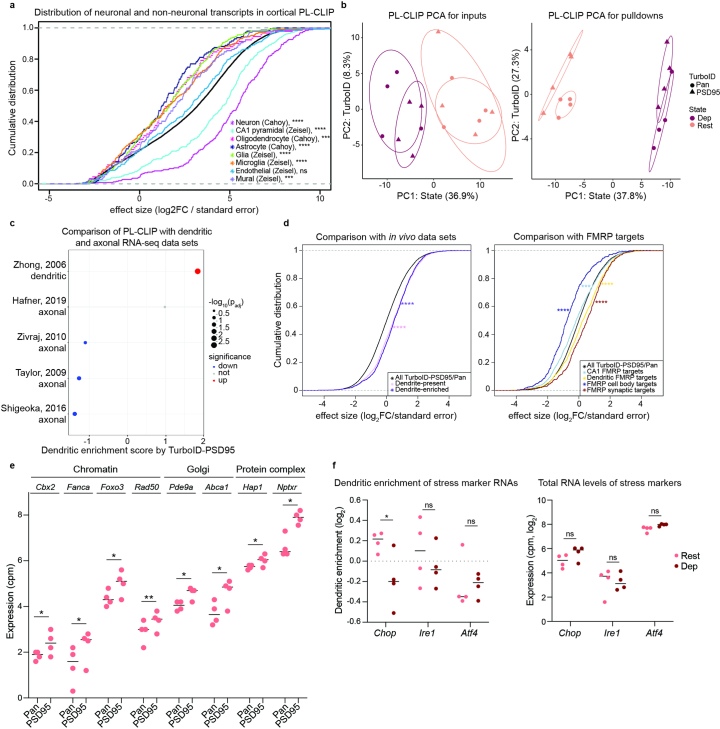
PL-CLIP identifies dendritic RNAs. **a**, The expression of neuronal (mouse CA1 pyramidal and developing forebrain) and non-neuronal (oligodendrocytes, astrocytes, glial cells, microglial cells, endothelial cells and mural cells) transcripts are assessed among RNAs detected by PL-CLIP. Significance was determined by the two-sided Wilcoxon signed-rank test. *P* values: Neuron and CA1 pyramidal < 2.2e^−16^, Oligodendrocyte = 0.00013, Astrocyte = 1.71e^−8^, Glia < 2.2e^−16^, Microglia = 2.77e^−12^, Endothelial = 0.21, Mural = 0.00043. **b**, Principal component analysis (PCA) shown for Pan-TurboID and TurboID-PSD95 inputs (left) and streptavidin pulldowns (right) in resting and depolarized PL-CLIP (*n* = 4 biologically independent samples). Inputs separate only by neuronal state but not by TurboID; whereas, pulldowns are differentiated by state and TurboID (localization). **c**, Comparison of PL-CLIP-enriched RNAs with previously published dendritic^29^ and axonal^36–39^ RNAs (FDR < 0.05). Multiple testing correction was performed using the Benjamini-Hochberg method. **d**, Cumulative distribution function (CDF) plot of PL-CLIP RNAs, dendrite-enriched/present RNAs (left) and FMRP CA1 targets (right) from^14^. FC: fold change. Significance was calculated by the two-sided Wilcoxon signed-rank test. *P* values: Dendrite present and enriched < 2.2e^−16^, CA1 FMRP targets = 0.00059, Dendritic FMRP targets = 3.69e^−10^, FMRP cell body targets < 2.2e^−16^, FMRP synaptic targets = 2.23e^−13^. **e**, Examples of dendritically-enriched RNAs that encode chromatin, Golgi and protein complex localization-related proteins (*n* = 4, values from PL-CLIP). *P* values: *Cbx* = 0.049, *Fanca* = 0.026, *Foxo3* = 0.012, *Rad50* = 0.0022, *Pde9a* = 0.019, *Abca1* = 0.032, *Hap1* = 0.035, *Nptxr* = 0.022. Significance was calculated using the two-tailed, paired Student’s *t*-test. **f**, Left: Dendritic enrichment of *Chop*, *Ire1* and *Atf4* levels in resting and depolarized neurons. Dendritic enrichment (log_2_) is calculated by normalizing the transcript levels in TurboID-PSD95 to those in Pan-TurboID. *P* values: *Chop* = 0.038, *Ire1* = 0.47, *Atf4* = 0.99. Right: Total *Chop*, *Ire1* and *Atf4* expression levels (log_2_ cpm) are shown in the inputs from resting and depolarized Pan-TurboID-transduced neurons (*n* = 4, values from PL-CLIP). *P* values: *Chop* = 0.14, *Ire1* = 0.95, *Atf4* = 0.052. Significance was calculated using the two-tailed, unpaired Student’s *t*-test. *P* values: ns (not significant) >0.05; * <0.05; ** <0.01; *** <0.001; **** <0.0001. *n* indicates the number of biologically independent samples.

To isolate local dendritic mRNAs enriched before and after depolarization, we extended previous CLIP studies^14,26,27^ by integrating TurboID proximity ligation (PL) and termed this strategy PL-CLIP. Biotin-labeled UV-crosslinked RNA–protein complexes in neurons expressing TurboID constructs were purified using streptavidin, and RNAs directly bound to the biotinylated proteome were purified. Crosslinked RNAs isolated by Pan-TurboID or TurboID-PSD95 were sequenced across four biological replicates in resting and KCl-depolarized neurons, which were deprived of contaminating non-neuronal cell types as assessed by IF and transcriptomic analyses (Extended Data Figs. 1a and 2a). Principal component analysis (PCA) revealed that depolarization state was the primary differentiator across replicates both for inputs and for pulldowns; however, TurboID (localization) was important only for pulldowns, indicating compartment-specific and activation-specific isolation (Extended Data Fig. 2b).

**Fig. 2:**
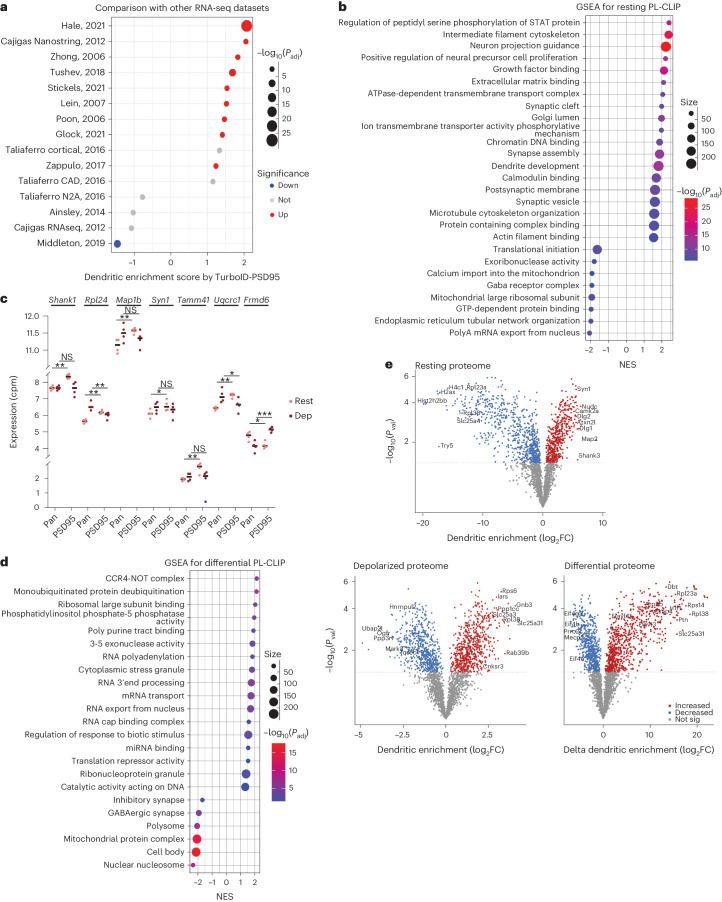
TurboID combined with CLIP and MS reveals compartment-specific changes of RNAs and proteins in resting and KCl-depolarized neurons. **a**, Comparison of PL-CLIP with other dendritic RNA-seq datasets (Methods; FDR < 0.05). **b**, GSEA on PL-CLIP-enriched transcripts in resting neurons (FDR < 0.05). **c**, Examples of PL-CLIP-enriched RNAs in resting and KCl-depolarized neurons. Expression (cpm) is plotted as log_2_ (*n* = 4 biologically independent samples, values from PL-CLIP). Colors: salmon (resting); burgundy (depolarized). Significance was calculated using the two-tailed, paired Student’s *t*-test. *P* values: NS (not significant) >0.05; * <0.05; ** <0.01; *** <0.001; **** <0.0001. *P* values: *Shank1* (rest = 0.0033, dep = 0.42), *Rpl24* (rest = 0.0061, dep = 0.0047), *Map1b* (rest = 0.0035, dep = 0.18), *Syn1* (rest = 0.027, dep = 0.18), *Tamm41* (rest = 0.0064, dep = 0.24), *Uqcrc1* (rest = 0.0024, dep = 0.019) and *Frmd6* (rest = 0.019, dep = 0.00081). **d**, GSEA on dendritically enriched RNAs upon depolarization (differential PL-CLIP: depolarized minus resting) (FDR < 0.05). **e**, Volcano plots showing proteins enriched and de-enriched (by log_2_ fold change (FC)) in dendrites according to resting (449 enriched; 835 de-enriched), depolarized (658 enriched; 594 de-enriched) and differential (depolarized minus resting) (808 enriched; 609 de-enriched) PL-MS (*n* = 5 biologically independent samples). Significance was calculated using the two-tailed, paired (resting and depolarized) and unpaired (differential) Student’s *t*-test. Multiple testing correction was performed using the Benjamini–Hochberg method (**a**,**b**,**d**). NES, normalized enrichment score.

The dendritic enrichment for each transcript was calculated by comparing RNAs crosslinked to TurboID-PSD95-labeled versus Pan-labeled proteins. The results revealed 2,788 and 3,727 transcripts enriched in resting and depolarized dendrites, respectively (Supplementary Table 1). PL-CLIP data from resting dendrites corresponded well with previously published datasets of neuropil transcripts (Fig. 2a)^1,9,10,14,28–35^. Moreover, previously established axonal transcripts^36–39^ were depleted or not significantly enriched in our dendritic transcriptome, indicating that PL-CLIP could distinguish subcellular transcriptomes with high specificity (Extended Data Fig. 2c). Comparing PL-CLIP to in vivo dendritic RNAs and FMRP RNA targets from mouse hippocampal CA1 neurons previously identified by our laboratory^14^ revealed them to be significantly enriched in the dendritic transcriptome (Extended Data Fig. 2d). Although FMRP binds RNAs across the whole neuron^14,40,41^, we found only its postsynaptic targets to be enriched in our data, further supporting the overrepresentation of FMRP targets in dendritic RNAs and the ability of PL-CLIP to distinguish subcellular transcriptomes.

To explore the roles encoded by dendritically enriched transcripts, we performed gene set enrichment analysis (GSEA) in resting dendrites. Gene sets associated with postsynaptic membrane, dendritic development and cytoskeleton were enriched, consistent with previous reports (Fig. 2b)^1,9^. Several transcripts known to be localized in resting dendrites were enriched, including *Shank1*, *Rpl24*, *Map1b* and *Syn1*, along with previously known (*Tamm41*) and undiscovered (*Uqcrc1*) mitochondrial RNAs^9,42^ and others (*Frmd6*) with yet to be identified dendritic functions (Fig. 2c). Some of these new RNAs encoded for proteins associated with Golgi, protein complex localization and chromatin binding (Extended Data Fig. 2e). Together, these data demonstrate that PL-CLIP specifically enriches for dendrite-specific RNAs, including previously known and unknown transcripts.

We next used PL-CLIP to identify dendritic RNAs after neuronal depolarization. RNAs encoding proteins involved in RNA processing, 3′ end RNA regulation, RNA transport and RNP granules were enriched in depolarized dendrites (Fig. 2d). The latter set was particularly intriguing because P-bodies and RNP granules disassemble upon synaptic stimulation^43^. In contrast, we found that stress marker transcripts were decreased or unchanged in dendrites upon depolarization (Extended Data Fig. 2f), supporting the conclusion that response to depolarization was distinct from a classical stress response. Moreover, we examined the distribution of dendritic RNAs upon depolarization. *Shank1*, *Rpl24*, *Map1b*, *Syn1*, *Tamm41*, *Uqcrc1* and *Frmd6* showed changes in dendritic enrichment in response to depolarization. For example, *Rpl24* and *Uqcrc1* became de-enriched; *Frmd6*, however, became more localized in dendrites (Fig. 2c). FRMD6 regulates actin dynamics^44^; hence, its preferential localization in dendrites suggests that cytoskeletal processes are modified in response to neuronal activation.

**Extended Data Fig. 3:**
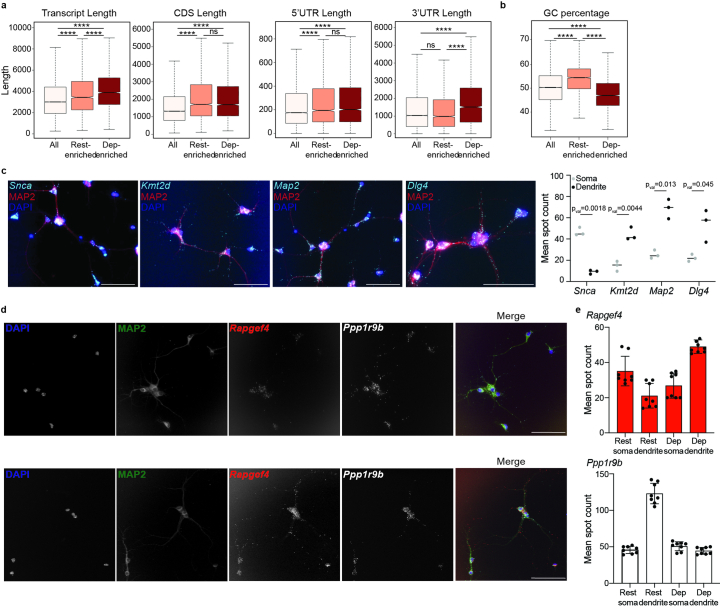
PL-CLIP identifies properties of dendritic RNAs in resting and depolarized neurons. **a**, Length and (**b**) GC content of resting and depolarized PL-CLIP-enriched dendritic RNAs and all detected RNAs in cortical neurons (‘All’, *n* = 17,654) are compared. Colors: salmon (resting, *n* = 2,788); burgundy (depolarized, *n* = 3,727). Significance was calculated by the two-sided Wilcoxon signed-rank test. Box plots show lower and upper hinges corresponding to the first and third quartiles (representing 25th and 75th percentile, respectively). Whiskers extend from the hinge to the 1.5x interquartile range. The center line indicates the median. *P* values: all significance values reported are < 2.2e-16 except for Transcript length (Rest vs. Dep = 4.18e-15), CDS (Rest vs. Dep = 0.081), 5′UTR (All vs. Rest = 2.10e-8; All vs. Dep = 4.22e-10; Rest vs. Dep = 0.93), 3′UTR (All vs. Rest = 0.15). **c**, RNA fluorescence in situ hybridization (RNA-FISH) combined with MAP2 IF to show dendrites and dendritically de-enriched (Snca) and enriched (Kmt2d, Map2 and Dlg4) RNAs by resting PL-CLIP. Images are quantified on the right (*n* = 3). Significance was derived from biological replicates, showing the center line at median and using the two-tailed, paired Student’s *t*-test. **d**, RNA-FISH combined with IF showing dendritic (MAP2-green) localization of Rapgef4 (red) and Ppp1r9b (white) in resting and depolarized neurons, (**e**) quantified for soma and dendrites. 4 fields of images per replicate for *n* = 2 presented as mean ±s.d. All images are taken at ×40 magnification, except for Dlg4, which was taken at ×60. *P* values: ns (not significant) >0.05; * <0.05; ** <0.01; *** <0.001; **** <0.0001. Scale bars, 100 μm. *n* indicates the number of biologically independent samples.

Our comparison of resting and depolarized transcriptomes revealed the properties of these dendritic transcripts. Overall, dendritic transcripts were longer in their CDSs and 5′ UTRs than the average lengths of all expressed transcripts, consistent with previous studies^14^ (Extended Data Fig. 3a). Although it was previously shown that transcripts enriched in neurites have longer 3′ UTRs^14^, we did not find this to be the case for PL-CLIP-enriched mRNAs in resting dendrites. This discrepancy might be explained by the specificity of our analysis, which is confined to interactions proximal to PSD95 in dendritic spines and not neurites. However, longer transcripts were enriched in dendrites after depolarization, and this difference in the length between resting and depolarized conditions was primarily driven by the 3′ UTRs (Extended Data Fig. 3a). Additionally, dendritic RNAs were richer in GC content in resting neurons, and this property switched upon activity (Extended Data Fig. 3b), potentially driven by significantly longer 3′ UTRs, which have lower GC content^45^. The presence of activity-related longer dendritic 3′ UTRs may reflect rapid recruitment of such transcripts to the PSD95 local subcompartment of the dendrite and allow synapses to decode genomic information by isoform selectivity. These 3′ UTRs might play diverse roles, ranging from regulation of local translation to recruitment of RBPs and generation of alternative protein-coding isoforms^46–49^.

To independently validate PL-CLIP in resting and activated neurons, we used RNAscope fluorescence in situ hybridization (FISH). We verified several dendritically enriched (*Kmt2d*, *Map2*, *Dlg4* and *Ppp1r9b*) and de-enriched (*Snca* and *Rapgef4*) RNAs that were identified in resting neurons in previous studies (Extended Data Fig. 3c,d). Upon activation, *Ppp1r9b* transcript levels decreased and were evenly distributed between soma and dendrites, whereas *Rapgef4* levels increased but became predominantly dendritic (Extended Data Fig. 3d,e).

**Extended Data Fig. 4:**
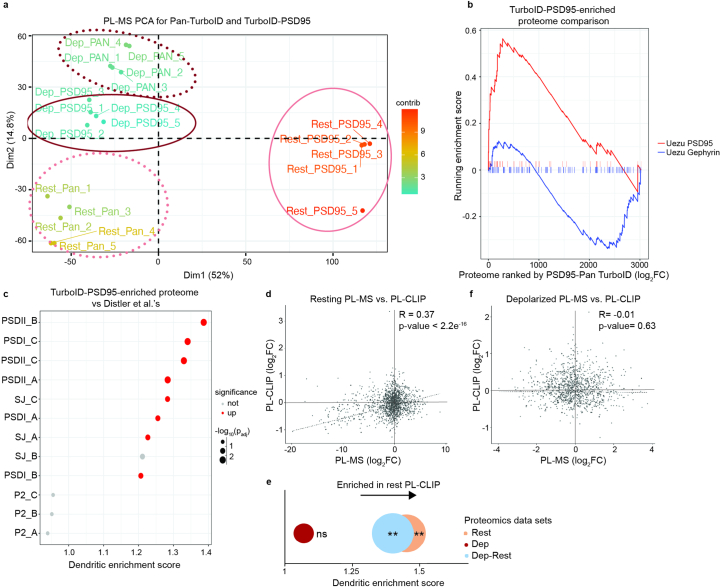
PL-MS identifies the dendritic proteome and its relation to local RNA levels. **a**, PCA plot showing five replicates of PL-MS from Pan-TurboID and TurboID-PSD95-transduced resting and depolarized neurons. Minus biotin counterpart of each sample is subtracted for each dot on the plot. **b**, Comparison of PL-MS-enriched proteome with previously published dataset^13^ (FDR < 0.05). **c**, TurboID-PSD95-enriched proteome vs. Distler et al. data^51^ (FDR < 0.05). Multiple testing correction was performed using the Benjamini-Hochberg method. **d**, Pearson correlation of PL-MS and PL-CLIP on dendritic RNAs in resting neurons. **e**, Shown are the distributions of PL-MS-enriched dendritic proteome from resting (rest), depolarized (dep) and differential (dep minus rest) data in ranked resting PL-CLIP using a GSEA-based strategy (FDR < 0.05) (Methods). Multiple testing correction was performed using the Benjamini-Hochberg method in the fgsea package. *P* adjusted: Rest = 0.0026, Dep = 0.18, Dep-Rest = 0.0027. *P* values: ** <0.01. **f**, Pearson correlation of PL-MS and PL-CLIP on dendritic RNAs in depolarized neurons. **(d,f)** The reported *P* values are two-sided.

To understand the extent to which the dendritically localized RNAs may determine local protein content, we performed mass spectrometry (MS) on the biotinylated proteome in resting and KCl-depolarized neurons. Because TurboID is a promiscuous biotin ligase, we used a ‘minus biotin’ condition for each sample, in which equivalent TurboID was expressed but no biotin was added, to subtract proteins labeled without temporal specificity (Fig. 2e). We defined dendritic localization of proteins by calculating their enrichment in TurboID-PSD95 over Pan-TurboID pulldowns after the ‘minus biotin’ counterpart of each was subtracted, and we termed this proximity ligation mass spectrometry (PL-MS) (Supplementary Table 2). All five replicates were consistent, with higher differential between the resting and depolarized conditions in the PSD95-enriched proteome than in the Pan-enriched proteome (Extended Data Fig. 4a).

We compared our resting proteomic results with a similar high-throughput approach employing a kinetically less efficient version of TurboID, BirA^R118G^, to isolate dendritic proteins in the mouse brain^13^. We specifically detected the reported PSD95-BirA proteome to be enriched and the inhibitory (Gephyrin-BirA) proteome to be depleted in our PL-MS (Extended Data Fig. 4b). We also looked at another dataset that used synaptosome preparation^50^ and found that this postsynaptic proteome had the strongest agreement with our PL-MS-enriched proteome (Extended Data Fig. 4c).

We then compared our PL-CLIP and PL-MS data and found that detected RNAs and proteins were highly correlated in resting dendrites (Extended Data Fig. 4d,e), but, surprisingly, this was not evident in depolarized conditions (Extended Data Fig. 4f).

### Dendritic TurboID identifies postsynaptic translatome

**Fig. 3:**
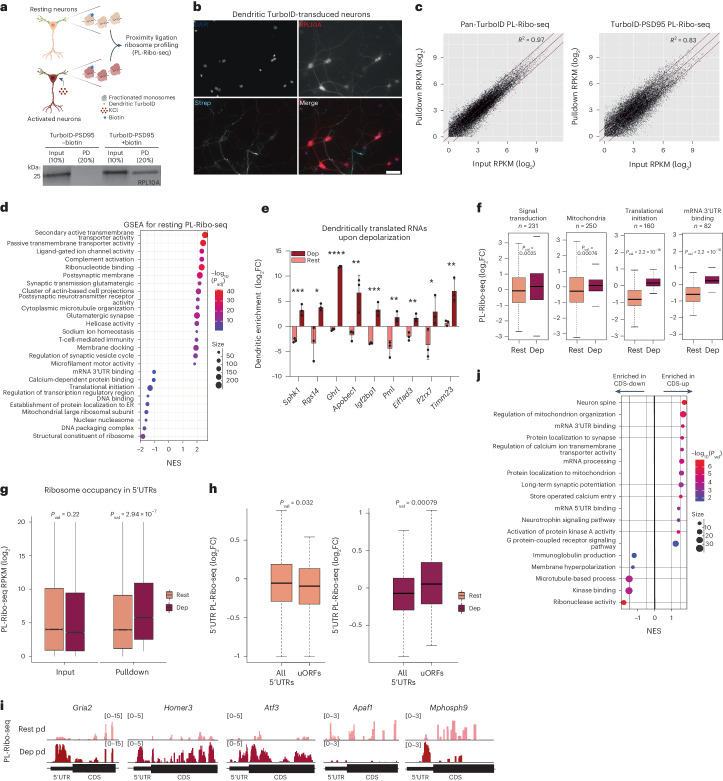
TurboID-mediated ribosome profiling reveals activity-dependent increase in ribosome occupancy and uORF usage in 5′ UTRs of dendritic mRNAs. **a**, Schematic for PL-Ribo-seq. TurboID-PSD95 labels dendritic ribosomes in primary cortical neurons. RPL10A western blot showing the input and streptavidin pulldown fractions from TurboID-PSD95-expressing neurons that are incubated with (+biotin) and without (−biotin) biotin for 30 min. Percentages refer to the volumetric percentage loaded on the gel. **b**, In TurboID-PSD95-transduced neurons, biotinylation (streptavidin, cyan) can be detected only in dendrites, even though RPL10A (red) stains across the whole neuron, as detected by IF. DAPI (blue) marker for nuclei. Magnification, ×20. Scale bar, 50 μm. **c**, Streptavidin pulldown and input RPKM values (log_2_) for each gene are plotted for Pan-TurboID (left) and TurboID-PSD95 (right) PL-Ribo-seq. Pan-TurboID pulldown and input correlation indicates that Pan-TurboID represents information from the whole neuron. **d**, GSEA on dendritically translated RNAs by resting PL-Ribo-seq (FDR < 0.05). Multiple testing correction was performed using the Benjamini–Hochberg method. **e**, Examples of RNAs that are translationally upregulated in dendrites in response to depolarization by PL-Ribo-seq (*n* = 3 biologically independent samples, values from PL-Ribo-seq). Dendritic log_2_ enrichment of translation for each RNA shown in resting and depolarized conditions. Significance was calculated using the two-tailed, unpaired Student’s *t*-test. Data are presented as mean ± s.d. *P* values: NS (not significant) >0.05; * <0.05; ** <0.01; *** <0.001; **** <0.0001. *P* values: *Spkh1* = 0.00046, *Rgs14* = 0.011, *Ghrl* = 7.12 × 10^−8^, *Apobec1* = 0.0075, *Igf2bp1* = 0.00053, *Pml* = 0.0023, *Eif1ad3* = 0.0022, *P2rx7* = 0.024 and *Timm23* = 0.0052. **f**, Examples of gene sets (*n* indicates number of genes detected in each gene set) that are translationally upregulated in dendrites with neuronal depolarization and that correspond to examples of RNAs from **e**. Significance for box plots was determined by the two-sided Wilcoxon signed-rank test. **g**, Ribosome occupancy in 5′ UTRs of all detected RNAs (*n* = 14,684 in rest; *n* = 14,770 in dep) in the whole neuron (input) and in dendrites (pulldown) detected by PL-Ribo-seq from TurboID-PSD95-transduced neurons. RPKM changes for each region between resting and depolarized conditions were tested using the permutation *t*-test. The *P* values were then adjusted with Bonferroni correction. **h**, Dendritic translation of 5′ UTRs with uORFs identified by ORF-RATER in resting and depolarized PL-Ribo-seq. Rest: *n* of all 5′ UTRs = 10,818; uORFs = 967. Dep: *n* of all 5′ UTRs = 10,718; uORFs = 859. Significance for box plots was determined by the one-sided Wilcoxon signed-rank test. **i**, Examples of RNAs with uORFs in their 5′ UTRs. PL-Ribo-seq reads (log_2_) in 5′ UTRs and CDS from the pulldowns (pd) of TurboID-PSD95-transduced neurons are shown for resting (top) and depolarized (bottom) dendrites. **j**, GSEA performed on translationally upregulated and downregulated dendritic RNAs with increased 5′ UTR translation in response to depolarization (*P* < 0.01). *P* values were derived using the ‘fgsea’ package and the adaptive multi-level split Monte Carlo method. Colors: salmon (resting); burgundy (depolarized). Box plots show lower and upper hinges corresponding to the first and third quartiles (representing the 25th and 75th percentiles, respectively). Whiskers extend from the hinge to the 1.5 × interquartile range. The center line indicates the median. FC, fold change; NES, normalized enrichment score. Source data

**Extended Data Fig. 5:**
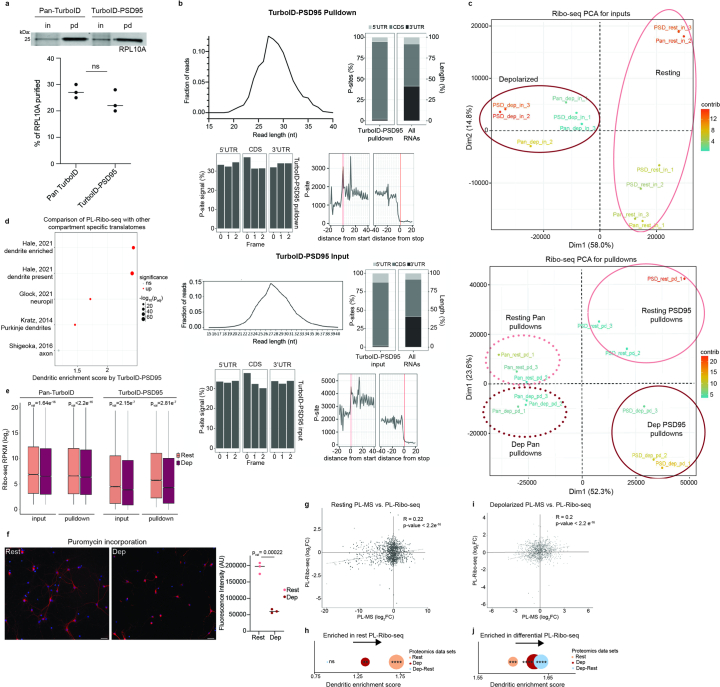
PL-Ribo-seq accurately identifies locally translated RNAs and their relation to local protein levels in dendrites. **a**, Percent RPL10A recovered from pulldown samples from Pan-TurboID or TurboID-PSD95-transduced neurons was calculated by dividing the pulldown band (100% volume loaded) value by that of the input band (10% volume loaded) (*n* = 3). The center line is at median. Significance was calculated using the two-tailed, paired Student’s *t*-test. **b**, Quality metrics of ribosome profiling data on the streptavidin pulldown fraction of TurboID-PSD95 in resting neurons shown on the top row. The coverage in the CDS and UTRs are shown. Similar metrics are shown for the input fraction in the bottom two rows: the read length distribution, percentage of P-sites in the CDS and UTRs along with the length of each region, the P-site signal in each reading frame and the P-site coverage from the start and stop codons. **c**, PCA shown for Pan-TurboID and TurboID-PSD95 input (left) and streptavidin pulldown (right) fractions in resting and depolarized neurons for three replicates of PL-Ribo-seq. **d**, Comparison of TurboID-PSD95-enriched translated RNAs in resting neurons with previously published sets of dendritic and axonal translatomes using a GSEA-based strategy. (FDR < 0.05). **e**, Ribosome profiling RPKM read distribution in the coding sequences (CDS) of all detected RNAs (n of Rest = 18,855, n of Dep = 18,887) in inputs and streptavidin pulldowns from Pan-TurboID and TurboID-PSD95-transduced resting and depolarized neurons. RPKM changes for each region between resting and depolarized conditions were tested using the permutation t-test. The *P* values were then adjusted with Bonferroni correction. Box plots show lower and upper hinges corresponding to the first and third quartiles (representing 25th and 75th percentile, respectively). Whiskers extend from the hinge to the 1.5x interquartile range. The center line indicates the median. **f**, Puromycin incorporation in resting and depolarized neurons shown by IF using a puromycin-specific antibody. DAPI (blue) marker for nuclei, red for puromycin. Magnification, ×20. Corrected fluorescence per cell was calculated by counting 45 cells (*n* = 3). Significance was derived from the biological replicate averages and calculated using the two-tailed, unpaired Student’s *t*-test. The center line is at median. Scale bars, 50 μm. **g**, Pearson correlation of PL-MS and PL-Ribo-seq on dendritic RNAs in resting neurons. **h**, Shown are the distributions of PL-MS-enriched dendritic proteome from resting (rest), depolarized (dep) and differential (dep minus rest) data in ranked resting PL-Ribo-seq using a GSEA-based strategy (FDR < 0.05). *P* adjusted: Rest = 3.70e^−9^, Dep = 0.0019, Dep-Rest = 0.69. **i**, Pearson correlation of PL-MS and PL-Ribo-seq on dendritic RNAs in depolarized neurons. **j**, Shown are the distributions of PL-MS-enriched dendritic proteome from resting, depolarized and differential data in ranked differential PL-Ribo-seq using a GSEA-based strategy (FDR < 0.05). *P* adjusted = Rest = 0.00083, Dep = 1.60e^−13^, Dep-Rest = 6.95e^−16^. *P* values: ** <0.01; *** <0.001; **** <0.0001. *n* indicates the number of biologically independent samples. **(d,h,j)** Multiple testing correction was performed using the Benjamini-Hochberg method. **(g,i)** The reported *P* values are two-sided. Source data

We were intrigued by the lack of correlation of PL-CLIP and PL-MS data in depolarized conditions and wanted to understand if rapid changes in local translation could shape the local proteome after depolarization. TurboID-PSD95 transduction of neurons resulted in biotinylation of dendritic proteins and ribosomes, as shown by western blots after streptavidin pulldowns (Fig. 3a), and IF of RPL10A, a component of the 60S ribosomal subunit, which co-localized with streptavidin (Fig. 3b). To monitor dendritic translation in the postsynaptic compartment, we combined TurboID labeling with ribosome profiling (deep sequencing of ribosome-protected mRNA fragments)^51^. Specifically, we purified biotinylated ribosomes from Pan-TurboID-expressing and TurboID-PSD95-expressing cells and used sequencing to quantitate ribosome-protected mRNA footprints in the input and pulldown fractions (Fig. 3a and Extended Data Fig. 5a). We calculated the dendritic translation of mRNAs as the ratio of ribosome protected footprints mapping to those in the PSD95 pulldown compared to input samples, and we termed this approach proximity ligation ribosome sequencing (PL-Ribo-seq) (Supplementary Table 3). The ribosome profiling protocol was optimized to accommodate the low input nature of our samples, and we applied riboWaltz^52^ and Plastid^53^ to assess quality control (Extended Data Fig. 5b). Three replicates were consistent across TurboID constructs and conditions (Extended Data Fig. 5c).

To determine whether ribosomes were labeled in a compartment-specific manner, the ribosome-protected fragments in pulldowns and inputs for Pan-TurboID and TurboID-PSD95 were analyzed. Although Pan-TurboID provides information from the whole neuron, TurboID-PSD95 targeted only dendritic ribosomes (Fig. 3c). PL-Ribo-seq agreed with previously published datasets on resting dendrite-specific but not axon-enriched translatomes (Extended Data Fig. 5d), allowing a comprehensive picture of translation specifically in the postsynaptic space.

In resting dendrites, PL-Ribo-seq identified gene sets that were also found in PL-CLIP, with roles in postsynapse, glutamatergic synapse, microtubule cytoskeleton and endosome, and was depleted in ones encoding nucleosome, DNA packaging, mitochondrial translation and translational regulation-related proteins (Fig. 3d). Additionally, although immunity-related transcripts were present only in resting dendrites, they were substantially enriched in the resting dendritic translatome (Fig. 3d), which was largely unanticipated despite prior evidence of the importance of class I major histocompatibility complex (MHC) molecules in remodeling synaptic connections^54,55^.

KCl-depolarized dendrites showed altered translation of specific mRNAs with diverse functions. *Sphk1*, *Rgs14* and *Ghrl*, with roles in G protein-coupled receptor signaling and postsynaptic chemical transmission; *Apobec1* and *Igf2bp1*, with roles in regulating binding at 3′ UTRs; *Pml* and *Eif1ad3*, associated with translation initiation; and *P2rx7* and *Timm23*, involved in ATP-coupled respiration and mitochondrial protein import, showed increased translation in dendrites in response to depolarization (Fig. 3e). These mRNAs were present in gene sets that were significantly overrepresented in the depolarized dendritic translatome (Fig. 3f).

To further understand activity-dependent local translational control, we analyzed global changes in translation upon depolarization. We observed a substantial decrease in the number of ribosomes found on the CDS of dendritic transcripts (Extended Data Fig. 5e) as well as of pan-neuronal transcripts (detected by Pan-TurboID, in agreement with previous reports)^56,57^. We extended these observations by finding reduced puromycin incorporation, a means of measuring active translation, in depolarized neurons (Extended Data Fig. 5f). Translational downregulation during neuronal depolarization might allow the recovery of energy and resources and/or reversion to a re-polarized state.

Comparing PL-Ribo-seq and PL-MS data revealed that PL-Ribo-seq correlated with the equivalent dendritic proteomics datasets in all conditions, in contrast to PL-CLIP (Extended Data Fig. 5g–j). These findings indicated that there is a discrepancy between the RNA levels and protein levels in dendrites, suggesting that ribosomal complexes are acutely rearranged after depolarization. Moreover, these findings indicate that localized translation is a more precise predictor of the local proteomic composition than simply the presence of RNAs, particularly in response to neuronal activity.

### Dendritic uORFs regulate a translational switch at downstream CDSs

**Extended Data Fig. 6:**
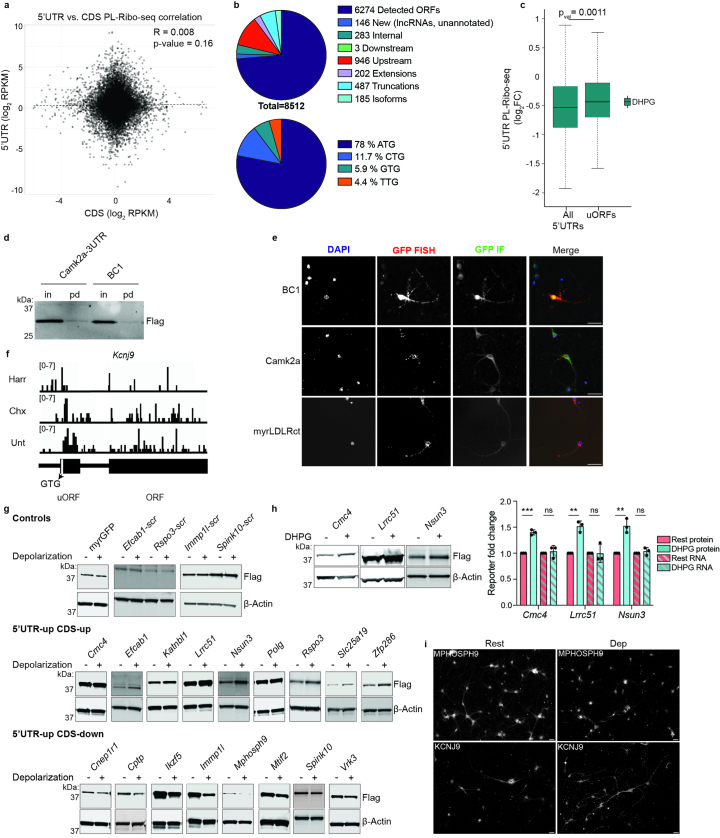
Dendritically localized reporters reveal the effects of 5′ UTRs on the downstream translation in an activity-dependent manner. **a**, Shown is the Pearson correlation of differential (depolarized minus resting) PL-Ribo-seq counts (log_2_ RPKM) from 5′UTR and CDS across all transcripts detected by dendritic ribosome profiling. Correlation coefficient, R, and the two-sided *P* value are shown in the upper right corner. **b**, Results of ORF-RATER analysis on all detected ORFs (top) and start codon usage (bottom). **c**, Dendritic translation of uORFs that are identified by ORF-RATER in DHPG-activated PL-Ribo-seq (*n* of all 5′UTRs = 10,313; uORFs = 881). Significance was calculated by the one-sided Wilcoxon signed-rank test. Box plots show lower and upper hinges corresponding to the first and third quartiles (representing 25th and 75th percentile, respectively). Whiskers extend from the hinge to the 1.5x interquartile range. The center line indicates the median. **d**, Different dendritic localization signals, Camk2a-3′UTR and BC1, are tested by streptavidin pulldowns from TurboID-PSD95 expressing cortical neurons. **e**, FISH and IF performed on superfolder GFP localized with BC1, Camk2a-3′UTR and myr-LDRct localization signals. DAPI shown for nuclei. For FISH and IF, GFP RNA and protein (by Flag antibody) are targeted, respectively. Magnification, ×20. Scale bars, 25 µm. **f**, ORF-RATER ribosome coverage in the 5′UTR and CDS of *Kcnj9*, with a non-cognate start codon in its uORF, GTG. Harr: harringtonine-treated; Chx: cycloheximide-treated; Unt: untreated neurons. **g**, Shown are western blots that are quantified in Fig. 4e and f. GFP protein detected by Flag, and β-Actin used as loading control. **h**, GFP fold changes of dendritic reporters with *Cmc4*, *Lrrc51* and *Nsun3* 5′UTRs are quantified by Flag and β-Actin western blots and qPCRs upon neuronal activation by DHPG (*n* = 3 biologically independent samples). All data are presented as mean ±s.d. Significance was calculated using the two-tailed, unpaired Student’s *t*-test. *P* values: *Cmc4* (protein = 0.00016; RNA = 0.64), *Lrrc51* (protein = 0.0010; RNA = 0.98), *Nsun3* (protein = 0.0027; RNA = 0.42). **i**, Larger fields of IFs for MPHOSPH9 (downregulated) and KCNJ9 (upregulated) in resting and depolarized (dep) neurons in support of Fig. 4i. Magnification, ×20. Scale bars, 25 µm. *P* values: ns (not significant) >0.05; * <0.05; ** <0.01; *** <0.001; **** <0.0001. Source data

Although our data and previous reports show that overall translation decreases upon neuronal depolarization^56,57^, they do not address if and how specific transcripts become translationally upregulated to meet synaptic needs. One of the most surprising findings from our data was that there was an increase in ribosome occupancy in the 5′ UTRs of dendritically localized mRNAs upon depolarization (Fig. 3g). This was specific to the dendritic compartment, because ribosome profiling of the total ribosome pool did not reveal any global changes in 5′ UTR ribosome occupancy (Fig. 3g). Interestingly, we did not observe any correlation between changes in translation of the 5′ UTR and CDS (Extended Data Fig. 6a).

To better understand how increased ribosome occupancy in the 5′ UTR impacts translation, we sought to identify the full set of translated uORFs in 5′ UTRs in primary cortical neurons using ORF-RATER^58^, an algorithm that identifies translated ORFs based on features of ribosome profiling data. This approach detected 946 translated uORFs that were largely unknown (Extended Data Fig. 6b and Supplementary Table 4). Most of these (78%) used the canonical ATG start codon, although a prominent subset used near-cognate start sites (Extended Data Fig. 6b).

Integration of the set of translated uORFs with PL-Ribo-seq revealed that neuronal activation, by both KCl depolarization and DHPG, led to an overall increase in uORF translation in dendrites (Fig. 3h and Extended Data Fig. 6c), whereas translation of their corresponding CDS varied among individual mRNAs. For example, translation of a previously identified uORF present in the *Gria2* 5′ UTR increased with depolarization, but this was not accompanied by a substantial change in the expression of the downstream CDS (Fig. 3i). *Homer3* and *Atf3*, by contrast, displayed increased uORF translation upon depolarization that was accompanied by a concomitant increase in CDS translation in dendrites (Fig. 3i). The 5′ UTR and CDS translation of *Apaf1* were both decreased by depolarization, and *Mphosph9* was an example where translation increased in the 5′ UTR but decreased in the CDS (Fig. 3i).

Interestingly, for most dendritic mRNAs with increased ribosome occupancy in their 5′ UTRs after depolarization, there was a corresponding increase in CDS translation without a significant change in transcript levels. These mRNAs encoded proteins involved in mitochondrial biology, long-term potentiation and cell signaling (Fig. 3j and Supplementary Table 5). A distinct set of these transcripts with increased 5′ UTR ribosome occupancy had decreased CDS translation without a significant decrease in mRNA levels. These encoded proteins function in the immune system, microtubule processes and kinase binding, indicating specific but differential regulation of functionally unique groups of dendritic mRNAs (Fig. 3j). These data suggest that depolarization caused an upregulation of ribosome occupancy in dendritic uORFs (~40% and 34% of 946 for KCl and DHPG, respectively), which leads to complex regulation of the downstream CDS translation.

**Fig. 4:**
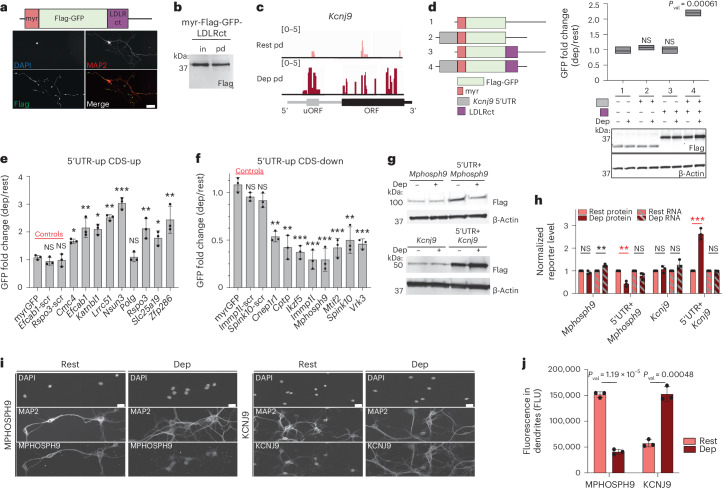
uORFs in the 5′ UTRs of dendritic mRNAs regulate downstream CDS translation upon neuronal activation. **a**, Dendritic GFP reporter with myristoylation (myr) and LDLR-C-terminal (LDLRct) sequences and its localization by Flag (green) IF. DAPI (blue) marker for nuclei; MAP2 (red) marker for dendrites. Magnification, ×20. Scale bar, 50 µm. **b**, Flag western blot for input (in) and pulldown (pd) from TurboID-PSD95-transduced neurons. **c**, Coverage of PL-Ribo-seq pulldown (log_2_) for *Kcnj9* uORF and main ORF from resting and depolarized (dep) TurboID-PSD95-transduced neurons. **d**, Myristoylated, myristoylated with *Kcnj9*-5′ UTR, myristoylated with LDLRct and myristoylated with LDLRct and *Kcnj9*-5′ UTR reporters in resting and depolarized neurons. Myristoylation and LDLRct sequences are in the CDS. GFP fold changes (dep/rest) from Flag (GFP) and β-Actin western blots are calculated as: [Flag^Dep^ / β-Actin^Dep^] / [Flag^Rest^ / β-Actin^Rest^]. All are compared to myrGFP negative control (*n* = 3). The center line is at mean. **e**,**f**, GFP fold changes of dendritic reporters with 5′ UTRs housing uORFs with increased translation upon depolarization (5′ UTR-up) and translationally upregulated (CDS-up) (**e**) or downregulated (CDS-down) (**f**) CDS. Negative controls: myrGFP, scrambled-*Efcab-*, -*Rspo3*-, -*Immp1l*- and -*Spink10*-5′ UTRs. All are compared to myrGFP (*n* = 4). *P* values: *Efcab1*-scr = 0.16; *Rspo3*-scr = 0.59; *Cmc4* = 0.013; *Efcab1* = 0.0067; *Katnb1l* = 0.021; *Lrrc51* = 0.0038; *Nsun3* = 0.00011; *Polg* = 0.89; *Rspo3* = 0.0086; *Slc25a19* = 0.011; *Zfp286* = 0.0089; *Immp1l*-scr = 0.089; *Spink10*-scr = 0.063; *Cnep1r1* = 0.0072; *Cptp* = 0.0021; *Ikzf5* = 0.00029; *Immp1l* = 0.00041; *Mphosph9* = 0.00067; *Mtif2* = 0.00077; *Spink10* = 0.0032; and *Vrk3* = 0.00031. **g**,**h**, Western blots of *Mphosph9* and *Kcnj9* CDS translation with and without their 5′ UTRs (**g**) are quantified by normalizing Flag expression in each condition to β-Actin levels (**h**). In the same plot, also shown are the qPCRs of reporters, normalized as described for the western blots (*n* = 3). Protein changes of CDS with 5′ UTRs are indicated by red asterisks. *P* values: *Mphosph9* (protein = 0.13; RNA = 0.0017), 5′ UTR + *Mphosph* (protein = 0.0014; RNA = 0.066), *Kcnj9* (protein = 0.49; RNA = 0.091), 5′ UTR + *Kcnj9* (protein =0.00031; RNA = 0.34). **i**,**j**, IF images (**i**) and quantifications (**j**) of MPHOSPH9 (downregulated) and KCNJ9 (upregulated) with MAP2 (dendrites) and DAPI (nuclei) in resting and depolarized neurons (*n* = 3). Magnification, ×20. Scale bars, 25 µm. All data are presented as mean ± s.d. Significance was calculated by two-tailed paired (**d**–**f**) and unpaired (**h**,**j**) Student’s *t*-test. *P* values: NS (not significant) >0.05; * <0.05; ** <0.01; *** <0.001; **** <0.0001. *n* indicates the number of biologically independent samples. Source data

Several studies established that uORFs can hamper or promote downstream CDS translation in different cell types, particularly under stress conditions; however, their roles in localized translational control or how they achieve this regulation have not been fully explored^59–62^. Previous studies of localized RNAs in dendrites paved the way for the discovery of a wide range of dendritic localization signals, mainly in 3′ UTRs of transcripts, termed zipcodes^63–69^. We used zipcodes to direct the localization of reporter mRNAs and, thereby, explore the impact of neuronal activity on dendritic translation. We tested the effects of three zipcodes—Camk2a-3′ UTR, BC1 and myristoylated-LDLR-C-terminal (myr-LDLRct) sequences—on the localization and dendritic protein accumulation of a reporter mRNA that encodes for Flag-tagged GFP^70^ in cortical neurons. We analyzed reporter mRNA localization by FISH and protein localization and dendritic translation via IF and TurboID-PSD95-mediated biotinylation. We found that myr-LDLRct most accurately localized the majority of reporter mRNA and protein to dendrites and allowed the reporter protein to be biotinylated by TurboID-PSD95 (Fig. 4a,b and Extended Data Fig. 6d,e), enabling us to study the effects of uORFs and other features of 5′ UTRs on activity-dependent postsynaptic CDS translation.

Using this reporter in cortical neurons, we tested the *Kcnj9-*5′ UTR, which harbors a uORF identified by ORF-RATER and showed increased 5′ UTR and CDS translation upon depolarization in dendrites by PL-Ribo-seq (Fig. 4c and Extended Data Fig. 6f). We detected a 2.5-fold increase in reporter protein levels after depolarization (Fig. 4d). This effect was strictly dependent on the dendritic localization and the 5′ UTR of the reporter mRNA (Fig. 4d), demonstrating that 5′ UTRs with uORFs are sufficient to confer activity-dependent translational control.

We next expanded the set of reporters using a selection of 17 5′ UTRs with uORFs based on the criteria that (1) they had increased ribosome occupancy in dendrites in response to depolarization; (2) they coincided with the greatest effects on levels of increased (Fig. 4e and Extended Data Fig. 6g) or decreased (Fig. 4f and Extended Data Fig. 6g) dendritic CDS translation without a significant change in their transcript levels (Supplementary Table 1); and (3) they were mostly uninvestigated (with the exception of *Polg* and *Immp1l* (refs. ^59,71^)). Strikingly, the downstream effects of 16 of 17 5′ UTRs tested agreed with the findings from PL-Ribo-seq on the endogenous genes that they were derived from. We further confirmed the activity-dependent increase in translation using DHPG activation for three 5′ UTR reporter constructs (*Cmc4*, *Lrrc51* and *Nsun3*) (Extended Data Fig. 6h). By contrast, scrambled versions of the same length 5′ UTRs failed to regulate translation, suggesting that specific motifs within these 5′ UTRs dictate downstream CDS translational regulation (Fig. 4e,f and Extended Data Fig. 6g).

To test whether the translational control observed with the GFP reporter accurately recapitulated control of the endogenous genes, we examined mRNAs in their natural contexts by replacing GFP with the *Mphosph9* or *Kcnj9* CDS, with and without their 5′ UTRs. *Mphosph9* and *Kcnj9* were examples of uORF-containing mRNAs with increased 5′ UTR translation in dendrites upon depolarization. The *Mphosph9* CDS was translationally downregulated (Fig. 3i), whereas *Kcnj9* was upregulated (Fig. 4c), by PL-Ribo-seq. Using the dendritic reporter, we found that the translation of *Mphosph9* decreased, and *Kcnj9* increased, only in the presence of their 5′ UTRs and, in both cases, did so independently of their mRNA levels in each condition (Fig. 4g,h). We were able to visualize the changes in the endogenous levels of these proteins in dendrites after depolarization by IF (Fig. 4i,j and Extended Data Fig. 6i), highlighting the robustness of our findings. These data further strengthened the observation that 5′ UTRs with uORFs are the necessary and independent units that drive changes in dendritic CDS translation after neuronal depolarization.

### eIF4G2 binds to 5′ UTRs and regulates local mRNA translation

**Fig. 5:**
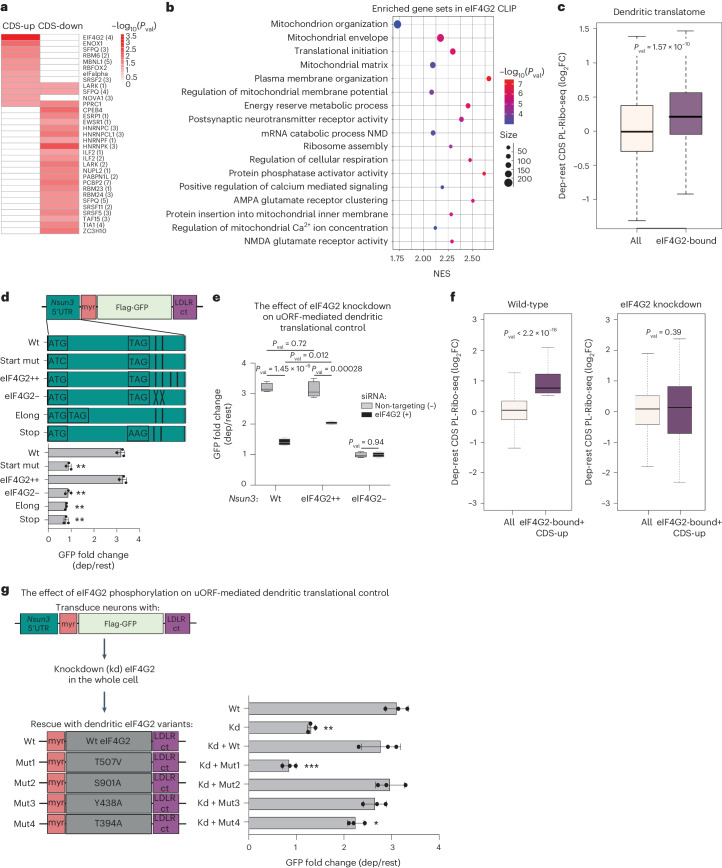
eIF4G2 is required to upregulate dendritic mRNA translation upon activation. **a**, Dendritic mRNAs with increased ribosome occupancy in their 5′ UTRs upon depolarization are divided into two groups: translationally upregulated (CDS-up) and downregulated (CDS-down) in their CDS. Enriched mRNA binding protein (RBP) sites in the 5′ UTRs of these groups are shown in the heatmap using motifs from RBPmap^72^. *P* values were determined using hypergeometric testing. **b**, GSEA performed on differential (depolarized minus resting) log_2_-ranked eIF4G2 CLIP target mRNAs bound in their 5′ UTRs. *P* values were derived using the ‘fgsea’ package and the adaptive multi-level split Monte Carlo method. NES, normalized enrichment score. **c**, mRNAs with increased eIF4G2 binding in their 5′ UTRs upon depolarization are determined by eIF4G2 CLIP and referred to as eIF4G2-bound. CDS translation of all detected mRNAs in the differential PL-Ribo-seq (depolarized (dep) minus resting (rest)) (all, *n* = 16,759) and the eIF4G2-bound group (*n* = 1,420) is compared. **d**, *Nsun3*-5′ UTR reporters: wild-type (Wt) harbors eIF4G2 binding sites (vertical bars); eIF4G2++, two more eIF4G2 binding sites added; eIF4G2−, endogenous eIF4G2 binding sites scrambled. uORF mutant *Nsun3*-5′ UTR reporters: Start mut (start codon mutated); Elong (stop codon inserted after start); Stop (stop codon mutated). Fold changes are quantified from western blots of Flag and β-Actin as in Fig. 4d, and significance was calculated by comparing to Wt (*n* = 3). *P* values: Start mut = 0.0022, eIF4G2++ = 0.59, eIF4G2− = 0.0023, Elong = 0.0010 and Stop = 0.0034. **e**, Western blot quantifications of Flag and β-Actin of *Nsun3*-5′ UTR reporters in non-targeting (−) or *eIF4G2* siRNA-treated (+) conditions in resting and depolarized neurons are quantified as in Fig. 4d (*n* = 4). Box plot whiskers extend to minimum and maximum, with the center line at median. Significance was calculated using the two-tailed, unpaired Student’s *t*-test. **f**, Subset of eIF4G2-bound mRNAs that are translationally upregulated (*n* = 321) are shown in Wt (all, *n* = 16,759) and eIF4G2 knockdown (all, *n* = 17,547) PL-Ribo-seq data, in both conditions compared to all detected mRNAs in the corresponding dep-rest PL-Ribo-seq. **g**, Dendritic translational regulation of the Wt *Nsun3*-5′ UTR reporter is tested in resting and depolarized Wt and eIF4G2 knockdown (Kd) neurons, in which eIF4G2 levels are rescued by a dendritically localized Wt or phospho-mutant version of eIF4G2. Fold changes from western blots of Flag and β-Actin are quantified as in Fig. 4d. Significance was calculated by comparing to Wt (*n* = 3). *P* values: Kd = 0.0090, Kd+Wt = 0.39, Kd+Mut1 = 0.00055, Kd+Mut2 = 0.66, Kd+Mut3 = 0.52 and Kd+Mut4 = 0.041. **c**,**f**, Significance was calculated using the two-sided Kolmogorov–Smirnov test. Box plots show lower and upper hinges corresponding to 25th and 75th percentiles. Whiskers extend from the hinge to the 1.5 × interquartile range. The center line indicates the median. **d**,**g**, Significance was calculated using the two-tailed, paired Student’s *t*-test. All data are presented as mean ± s.d. *P* values: NS (not significant) >0.05; * <0.05; ** <0.01; *** <0.001; **** <0.0001. *n* indicates the number of biologically independent samples.

**Extended Data Fig. 7:**
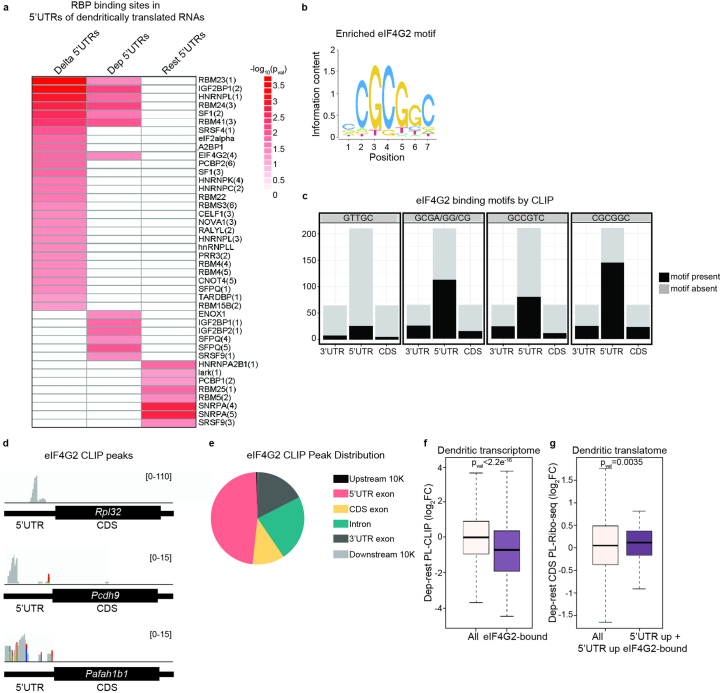
eIF4G2 binding increases in the 5′ UTRs of dendritic mRNAs upon depolarization and is associated with their increased translation. **a**, RBP sites in the 5′ UTRs of mRNAs enriched in resting (rest) and depolarized (dep) dendrites are shown in the heatmap using motifs from RBPmap^73^. *P* values were determined using hypergeometric testing. The RBP sites enriched more upon depolarization are shown in the delta (dep minus rest) column. **b**, The most enriched binding motif of eIF4G2 in 5′ UTRs that are increasingly translated in dendrites upon depolarization by PL-Ribo-seq. eIF4G2 has 4 predicted motifs in RBPmap. **c**, The distribution of eIF4G2 CLIP peak counts in 3′ UTRs, 5′ UTRs and CDS for all 4 eIF4G2 binding motifs. **d**, Examples of eIF4G2 target mRNAs identified in resting eIF4G2 CLIP (peak heights, log_2_); peaks in similar genomic locations were identified in human cell lines by eCLIP^82^. *Pcdh* and *Pafah1b1* harbor uORFs in their 5′ UTRs. **e**, The distribution of eIF4G2 CLIP peaks is calculated by combining the eIF4G2 peaks from resting and depolarized primary cortical neurons. **f**, mRNAs with 5′ UTRs that are increasingly bound by eIF4G2 upon depolarization as determined by eIF4G2 CLIP are referred to as eIF4G2-bound. All detected mRNAs in the differential (depolarized (dep) minus resting (rest)) PL-CLIP (All, *n* = 17,654) are compared to the eIF4G2-bound group (*n* = 1,413). **g**, Differential dendritic CDS translation of all mRNAs with increased ribosomes in their 5′ UTRs (All 5′UTR up, *n* = 1,920) and differential dendritic CDS translation of mRNAs with increased eIF4G2 binding and increased ribosome occupancy in their 5′ UTRs (5′UTR up + eIF4G2-bound, *n* = 238) are compared. The latter group is translated more in dendrites upon depolarization compared to the average of all mRNAs with increased ribosome occupancy in their 5′UTRs in dendrites. **(f,g)** Significance was calculated using the two-sided Kolmogorov-Smirnov test. Box plots show lower and upper hinges corresponding to the first and third quartiles (representing 25th and 75th percentile, respectively). Whiskers extend from the hinge to the 1.5x interquartile range. The center line indicates the median. *P* values: ns (not significant) >0.05; * <0.05; ** <0.01; *** <0.001; **** <0.0001.

Next, we sought to dissect mechanistically how uORFs might regulate CDS translation in an activity-dependent and localization-dependent manner. We hypothesized that RBPs might mediate the effects of uORFs on downstream translation. To that end, we examined RBP binding sites enriched in the 5′ UTRs of dendritically translated messages using RBPmap^72^. Comparing resting, KCl-depolarized and differential (depolarized minus resting) ribosome occupancy in the 5′ UTRs of all dendritically translated mRNAs that we discovered by PL-Ribo-seq, we found binding sites for IGF2BP1, HNRNPK/L, eIF2α and eIF4G2 to be enriched in the 5′ UTRs of all mRNAs that were translationally regulated after depolarization (Extended Data Fig. 7a). We further divided the group of dendritic mRNAs with enhanced 5′ UTR translation according to their downstream CDS translation properties (CDS-up versus CDS-down) to assess whether specific RBPs were associated with differential effects. HNRNPK and TIA1 binding sites were enriched in the 5′ UTRs of the CDS-down group (Fig. 5a), in agreement with their known roles in translational repression by binding at the 5′ UTRs^73,74^. In contrast, eIF4G2 and RBFOX2 binding sites were enriched only in the 5′ UTRs of the CDS-up group in dendrites (Fig. 5a).

Out of the four predicted binding motifs for eIF4G2, ‘CGCGGC’ (eIF4G2(4)) was the most enriched motif in the 5′ UTRs of translationally upregulated dendritic mRNAs (Fig. 5a and Extended Data Fig. 7b). Although the presence of translated uORFs canonically leads to decreased translation of downstream CDS, there are a few examples where they also enhance it^60,61,75^. The non-canonical translation initiation factor eIF4G2 is known to play roles in cap-dependent and cap-independent translation and to upregulate translation of RNAs with structured 5′ UTRs or uORFs in non-neuronal systems^76–79^. Previously known roles of eIF4G2 in translation initiation^80^ led us to focus on eIF4G2 as a potential activity-dependent translational regulator in dendrites.

We experimentally determined how neuronal activity might impact eIF4G2 binding by performing eIF4G2 CLIP in resting and KCl-depolarized primary cortical neurons. The ‘CGCGGC’ motif was the most crosslinked motif in our eIF4G2-CLIP (75% of all 5′ UTR peaks; Extended Data Fig. 7c), consistent with our analysis of transcripts undergoing translational control in dendrites (Fig. 5a). For the resting eIF4G2 CLIP, we found examples of bound RNAs previously identified in human cell lines^81^ (Extended Data Fig. 7d). Overall, approximately 50% of our eIF4G2 CLIP binding was localized in 5′ UTRs (Extended Data Fig. 7e), consistent with its purported function in translation initiation. Together, these data strongly implicated eIF4G2 binding in translational control after depolarization.

Using our CLIP data, we identified a set of mRNAs with increased eIF4G2 binding in their 5′ UTRs in response to depolarization (termed ‘eIF4G2-bound’; Supplementary Table 6). The eIF4G2-bound targets were particularly enriched in mitochondria-related mRNAs by GSEA, along with additional targets encoding signaling, receptor clustering and mRNA processing-related messages (Fig. 5b). We then examined the dendritic translation and localization of this group of mRNAs using our PL-Ribo-seq and PL-CLIP datasets. Transcripts that had more eIF4G2 binding in their 5′ UTRs were translationally upregulated in depolarized dendrites, despite the observation that their transcript levels were decreased (Fig. 5c and Extended Data Fig. 7f). Furthermore, eIF4G2-bound targets with increased 5′ UTR translation upon depolarization showed higher levels of CDS translation compared to all mRNAs with enhanced 5′ UTR translation in dendrites (Extended Data Fig. 7g). Overall, these analyses revealed that eIF4G2 binding at the 5′ UTRs is associated with enhanced translation independent of mRNA levels in response to neuronal activity in dendrites.

**Extended Data Fig. 8:**
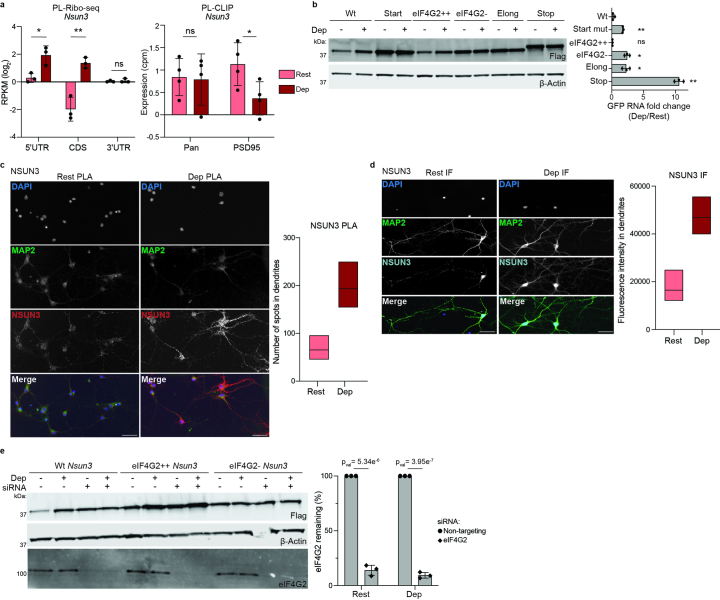
*Nsun3* is translationally upregulated in dendrites upon depolarization. **a**, Shown on the left is the dendritic translation by PL-Ribo-seq (log_2_) in the 5′ UTR, CDS and 3′ UTR of *Nsun3* in resting and depolarized neurons. Shown on the right are the PL-CLIP expression values (log_2_) of *Nsun3* in the Pan-TurboID and TurboID-PSD95 pulldowns from resting and depolarized neurons (*n* = 3, values from PL-Ribo-seq). *P* values: PL-Ribo-seq (5′UTR = 0.019; CDS = 0.0035; 3′UTR = 0.64), PL-CLIP (Pan = 0.88, PSD95 = 0.045). **b**, Protein and RNA analyses of *Nsun3*-5′UTR reporters in resting (Dep-) and depolarized (Dep+) neurons. Wild type (Wt) *Nsun3*-5′UTR harbors eIF4G2 binding sites; eIF4G2++, additional eIF4G2 binding sites; eIF4G2-, endogenous eIF4G2 binding sites scrambled. uORF mutants: Start mut (start codon mutated); Elong (stop codon inserted after start); Stop (stop codon mutated). Shown on the left are the Flag and β-Actin western blots that are quantified in Fig. 5d. Shown on the right are the qPCR quantifications (quantified as in Fig. 4d) for each *Nsun3* reporter (*n* = 3). Significance was calculated by comparing to the Wt and using the two-tailed, paired Student’s *t*-test. *P* values: Start mut = 0.0098, eIF4G2 + + = 0.26, eIF4G2- = 0.014, Elong = 0.048, Stop = 0.0026. **c**, Nascent translation (by proximity ligation assay, PLA) and **(d)** total protein (by IF) levels of NSUN3 are shown (*n* = 2) plotted as floating box plots from max to min with center lines at mean). Magnification, ×20. Scale bars, 25 µm. **e**, Western blots of Flag and β-Actin of *Nsun3*-5′UTR reporters in non-targeting (−) or *eIF4G2* siRNA-treated (+) conditions in resting and depolarized neurons for Fig. 5e. Western blot of eIF4G2 and the quantifications of the knockdowns are shown (*n* = 3). **(a,e)** Significance was calculated using the two-tailed, unpaired Student’s *t*-test. **(a,b,e)** All data are presented as mean ±s.d. *P* values: ns (not significant) >0.05; * <0.05; ** <0.01; *** <0.001; **** <0.0001. *n* indicates the number of biologically independent samples. Source data

We next studied the regulation of *Nsun3* as its transcript harbored an activity-dependent uORF that had the largest effect on downstream CDS translation (~3.3-fold increase; Fig. 4e) and had eIF4G2 binding sites in its 5′ UTR. Moreover, *Nsun3* plays a role in mitochondrial translation, consistent with our observation that mitochondrial regulation was the most enriched category of dendritic mRNAs upon depolarization (Figs. 3j and 5b). The absolute level of *Nsun3* CDS translation increased in depolarized dendrites by PL-Ribo-seq despite the fact that the local transcript levels were decreased (as measured by PL-CLIP; Extended Data Fig. 8a). *Nsun3*-5′ UTR has two endogenous eIF4G2 binding sites, and we generated reporters with a variety of mutations to study if uORF translation and/or eIF4G2 binding were necessary to enhance CDS translation (Fig. 5d). Wild-type *Nsun3* uORF and *Nsun3* uORF with two additional eIF4G2 binding sites (eIF4G2++) performed similarly and increased the downstream GFP translation approximately 3.3-fold without impacting mRNA levels (Fig. 5d and Extended Data Fig. 8b). However, translational increase was ablated by mutants that prevented uORF translation, despite an increase in mRNA levels. Moreover, eliminating all eIF4G2 binding sites (eIF4G2−) entirely abrogated depolarization-dependent upregulation of CDS translation, indicating that uORF translation along with eIF4G2 binding are both necessary for the downstream CDS upregulation.

Using a proximity ligation assay (PLA) that incorporates puromycin to visualize nascent translation and IF to assess total protein levels, we were able to confirm endogenous *Nsun3* regulation in resting and depolarized neurons. In agreement with our PL-Ribo-seq, we saw a significant increase in newly translated *Nsun3* in dendrites with depolarization along with an increase in total protein levels (Extended Data Fig. 8c,d). Notably, this activity-dependent increase in translation is eIF4G2 dependent, because siRNA-mediated knockdown of eIF4G2 or mutation of its binding sites (eIF4G2−) inhibited translational upregulation (Fig. 5e and Extended Data Fig. 8e).

**Extended Data Fig. 9:**
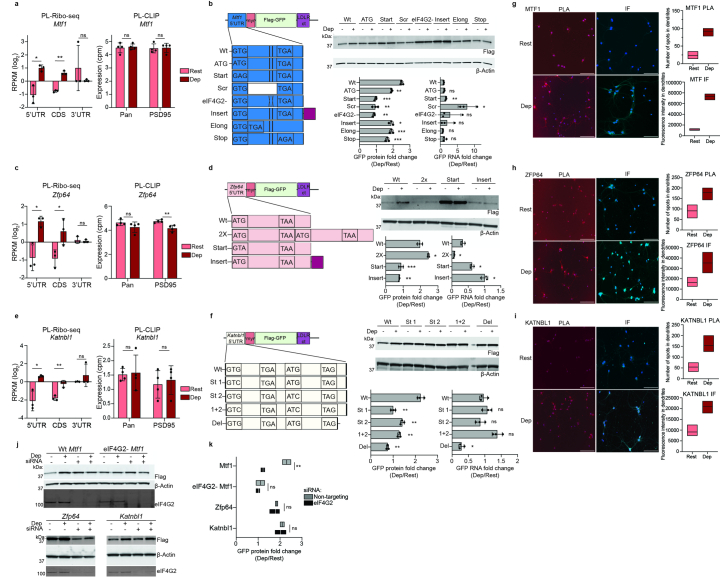
Activity-dependent eIF4G2 regulation by uORFs is specific to dendritic mRNAs with eIF4G2 binding sites in their 5′ UTRs. **a**, Shown on the left is the dendritic translation by PL-Ribo-seq (log_2_) in the 5′ UTR, CDS and 3′ UTR of *Mtf1* in resting and depolarized neurons (*n* = 3). Shown on the right are the PL-CLIP expression values (log_2_) of *Mtf1* in the Pan-TurboID and TurboID-PSD95 pulldowns from resting and depolarized neurons (*n* = 4). *P* values: PL-Ribo-seq (5′UTR = 0.0079; CDS = 0.00014; 3′UTR = 0.39), PL-CLIP (Pan = 0.74; PSD95 = 0.87). **b**, Reporter constructs with wild type (Wt) *Mtf1* 5′UTR, which have eIF4G2 binding sites (black vertical bars), and with *Mtf1* 5′UTR with mutations in its uORF. ATG, uORF start codon mutated to ATG; Start, uORF start codon mutated to GAG; Scr, uORF sequence scrambled with start and stop codons unchanged; eIF4G2-, eIF4G2 binding sites scrambled; Insert, distance between the uORF and GFP increased by randomized 15 nucleotide insertion; Elong, stop codon inserted after the start codon in the uORF; Stop, stop codon in the uORF is mutated to prevent uORF termination. The quantifications of the GFP protein and mRNA fold changes by western blots and qPCRs, respectively, are calculated and shown as in Fig. 4d (*n* = 3). *P* values: protein (Atg = 0.0091; Start = 0.00028; Scr = 0.0026; eIF4G2- = 0.0012; Insert= 0.029; Elong = 0.00073; Stop = 0.00046), RNA (Atg = 0.19; Start = 0.0092; Scr = 0.044; eIF4G2- = 0.43; Insert = 0.49; Elong = 0.065; Stop = 0.37). **(c**, **d)** Similar to **(a)** and **(b)**, PL-Ribo-seq and PL-CLIP data for *Zfp64* and the constructs with *Zfp64* 5′UTR (*n* = 3) are shown. *P* values: PL-Ribo-seq (5′UTR = 0.011; CDS = 0.040; 3′UTR = 0.66), PL-CLIP (Pan = 0.18; PSD95 = 0.0069). *Zfp64* 5′UTR does not have eIF4G2 binding sites. For *Zfp64*, a variant version of the uORF, where it is duplicated, is included (2x) as well as Start and Insert mutants designed as in **(b)**. *P* values: protein (2x = 0.047; Start = 0.00039; Insert = 0.0049), RNA (2x = 0.016; Start = 0.043; Insert = 0.019). **(e**, **f)** Similar to **(a)** and **(b)**, PL-Ribo-seq and PL-CLIP data for *Katnbl1* and the constructs with *Katnbl1* 5′UTR (*n* = 3) are shown. *P* values: PL-Ribo-seq (5′UTR = 0.013; CDS = 0.0037; 3′UTR = 0.38), PL-CLIP (Pan = 0.86; PSD95 = 0.68). Since *Katnbl1* uORF has two overlapping ORFs, separate start codon mutations (St 1 and St 2) as well as a double mutant (1 + 2) are included. Deletion (Del) mutant: the distance between the uORF and GFP start codon is decreased by 30 nucleotides. *Katnbl1* 5′UTR does not have eIF4G2 binding sites. *P* values: protein (St 1 = 0.0029; St 2 = 0.0057; 1 + 2 = 0.0062; Del = 0.0061), RNA (St 1 = 0.35; St 2 = 0.13; 1 + 2 = 0.085; Del = 0.022). **(g-i)** Nascent translation and total protein levels using PLA and IF, respectively, shown for G) MTF1, H) ZFP64 and I) KATNBL1. Magnification, ×20. Quantifications are shown on the right (*n* = 2) plotted as floating box plots from max to min with center lines at mean. **j**, Western blots showing GFP translation (Flag) from constructs with wild type and eIF4G2- *Mtf1* 5′UTR, *Zfp64* 5′UTR and *Katnbl1* 5′UTR with (siRNA+) and without eIF4G2 (siRNA-) knockdown. β-Actin used as loading control; eIF4G2 shown to indicate knockdowns. **k**, Flag and β-Actin western blots from **(j)** are quantified as in Fig. 4d. Significance was calculated between siRNA- and siRNA+ conditions (*n* = 3). The center lines for box plots are at mean. *P* values: *Mtf1* = 0.0013, eIF4G2- *Mtf1* = 0.55, *Zfp64* = 0.72, *Katnbl1* = 0.65. **(a,c,e,k)** Significance was calculated using the two-tailed, unpaired Student’s *t*-test. **(b,d,f)** All data are presented as mean ±s.d. Significance was calculated with respect to Wt, using the two-tailed, paired Student’s *t*-test. *P* values: ns (not significant) >0.05; * <0.05; ** <0.01; *** <0.001; **** <0.0001. *n* indicates the number of biologically independent samples. Source data

**Extended Data Fig. 10:**
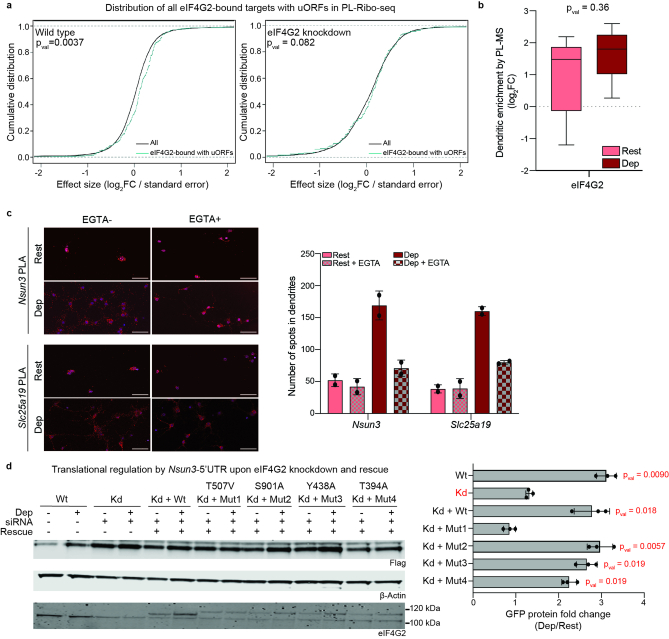
Translational upregulation of dendritic downstream ORFs by eIF4G2 is mediated by its local calcium-influx-induced phosphorylation. **a**, CDF plots of dendritic translation of eIF4G2-bound transcripts that harbor uORFs in wild type and eIF4G2 knockdown conditions. Significance was calculated by the two-sided Wilcoxon signed-rank test. **b**, Dendritic enrichment (log_2_) of eIF4G2 is shown using the resting and depolarized PL-MS data (*n* = 5, values from PL-MS data). Significance was calculated using the two-tailed, unpaired Student’s *t*-test. Box plot whiskers extend from min to max, with the center line at median. **c**, Nascent translation of *Nsun3* and *Slc25a19*, examples of nuclear-encoded mitochondria-related RNAs, is shown by PLA (red) in the presence (EGTA-) and upon depletion (EGTA+) of calcium in resting and KCl-depolarized neurons (*n* = 2). DAPI (blue, marker for nuclei). **d**, Shown are the Flag and β-Actin western blots that are quantified in Fig. 5g. eIF4G2 western blot shows endogenous eIF4G2 (lower bands), which is reduced by siRNA knockdowns (Kd), as well as the localized wild type and phospho-mutant eIF4G2 variants (upper bands, higher molecular weight due to the inclusion of dendritic localization signals). Fold changes are shown as in Fig. 5g but here significance was calculated with respect to the knockdown, using the two-tailed, paired Student’s *t*-test (*n* = 3). **(c,d)** All data are presented as mean ±s.d. *n* indicates the number of biologically independent samples. Source data

We observed additional transcripts that were translationally upregulated in dendrites through activity-induced uORF translation without a change in transcript levels (Extended Data Fig. 9a–i). Only uORFs with eIF4G2 binding sites, similar to that of *Nsun3*, were dependent on eIF4G2 to upregulate their CDS (for example, *Mtf1*; Extended Data Fig. 9j,k). In addition, eIF4G2-bound dendritic mRNAs that were translationally upregulated and harbored uORFs showed a significant decrease in activity-dependent local translation levels upon eIF4G2 knockdown (Fig. 5f and Extended Data Fig. 10a). These findings further establish that dendritic eIF4G2 binding within the 5′ UTRs is necessary for the activity-dependent increase in the translation of the main open reading frame by uORFs.

To assess how eIF4G2 might mediate its activity-dependent translational control, we measured total eIF4G2 protein in resting and KCl-depolarized dendrites. Although there was a slight increase in total eIF4G2, it was not significant (Extended Data Fig. 10b). We, thus, hypothesized that eIF4G2 phosphorylation might be critical for its activity-dependent role because another eIF4G protein, eIF4G3, has been shown to be a target of calcium-dependent phosphorylation^82^, and we found calcium influx upon depolarization (Fig. 1e and Extended Data Fig. 1m) to be critical for the translational changes that we observed (Extended Data Fig. 10c). To that end, we engineered dendritically localized wild-type and phospho-mutant versions of eIF4G2 using our dendritic localization signals (Figs. 4a and 5f and Extended Data Fig. 10d) and rescued eIF4G2 levels with these variants where eIF4G2 was depleted across the whole neuron. Although all eIF4G2 mutants accumulated at similar levels as the wild-type protein (Extended Data Fig. 10d), only T507V mutant completely failed to rescue activity-dependent translational control, suggesting that this site might be critical for calcium-mediated regulation of dendritic protein synthesis by eIF4G2.

## Discussion

Activity-dependent alterations in RNA localization and localized translation in synapses underlie learning and memory^3,6^; however, to date, our ability to globally analyze the mechanisms and dynamics of their regulation has been limited. Here we describe a new proximity-based labeling platform engineered to monitor activity-dependent changes in postsynaptic mRNA, translation and protein levels. This approach revealed a new mechanism of translational control in which depolarization leads to calcium influx, eIF4G2 phosphorylation and de novo binding to target transcripts and increased uORF translation in the 5′ UTRs of those transcripts, resulting in enhanced downstream protein synthesis. Our studies provide an unprecedented view into both the nature and mechanisms by which neuronal activity sculpts the dendritic proteome.

KCl treatment was initially chosen for triggering neuronal depolarization because it is well established, has been widely used to study synaptic biology and enables the detection of robust changes in dendrites. We recognize that prolonged KCl activation does not mimic physiologic neuronal stimulation. To demonstrate the rigor of our experimental findings, we shortened the KCl activation time, relative to previous prolonged depolarizations^83–85^, and, notably, replicated our key findings with the glutamate agonist DHPG. Although dephosphorylation followed by increased de novo translation has been observed in behavioral studies^86,87^, our finding that depolarization induces phosphorylation of translation control factors and global translational downregulation agrees with several other analyses^88–92^. The disparity among studies could be attributed to different activation paradigms and experimental strategies or could, in fact, highlight the spatial and temporal complexity of activity-dependent synaptic translational control.

Our results indicate that, although multiple mechanisms contribute to activity-dependent changes in dendritic proteomes, translational control is crucial in regulating the rapid upregulation of a functionally coherent subset of messages in dendrites. This finding helps elucidate one of the longstanding conundrums in the field: why do dendrites localize a large number of mRNAs when many of these messages are poorly engaged by the translation machinery?

**Fig. 6:**
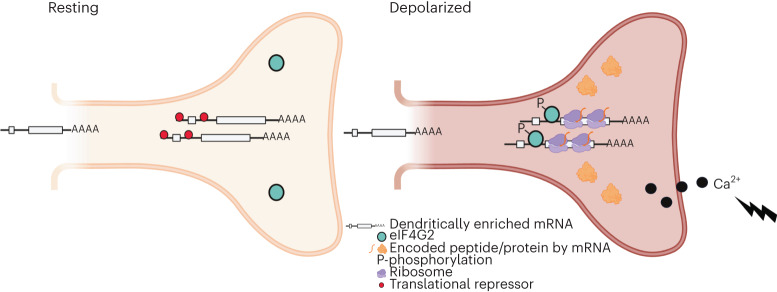
Model for activity-dependent eIF4G2:uORF-mediated translational control in dendrites. A subset of dendritically enriched mRNAs, including those with roles in signaling and mitochondrial functions, is translationally silent in resting postsynaptic sites. We demonstrate that, after neuronal activation and calcium influx, eIF4G2 is phosphorylated at threonine (T507), enabling direct binding to select 5′ UTRs (Fig. 5g) to upregulate the translation of the downstream CDSs. Translation of uORFs from these 5′ UTRs is enhanced even though overall dendritic translation is suppressed (Fig. 3h and Extended Data Figs. 5e and 6c). eIF4G2 binding then allows efficient scanning of ribosomes into the downstream CDSs, enabling the rapid production of dendritic proteins that are needed for synaptic plasticity and energy homeostasis.

To address this unknown, we compared RNA, translation and protein levels in resting and activated dendrites treated with short pulses of either KCl or DHPG, accompanied by calcium influx. The lack of correlation in RNA and protein levels upon depolarization led us to examine the translation of pre-existing pools of localized RNAs and to uncover a uORF-mediated mechanism of protein synthesis. These pre-emptively localized RNAs contain necessary information in their 5′ UTRs to allow for activity-dependent uORF translation and recruitment of phosphorylated eIF4G2 that, together, upregulate downstream ribosome binding and CDS translation (Fig. 6). Additionally, we noted the differences in the resting basal protein levels when the 5′ UTRs cannot be translated or bound by eIF4G2, which might be driven by changes in RNA levels (Extended Data Fig. 8b,e). We postulate that this involves additional *cis*-acting signals on these transcripts. Taken together, this mechanism brings a new understanding as to why these RNAs are enriched at postsynaptic sites and how eIF4G2:uORF targeting can rapidly change ribosome scanning to generate a coherent set of proteins upon synaptic needs.

Neuronal survival and synaptic and metabolic plasticity depend critically on ATP production and calcium buffering by mitochondria^93^. The changes in protein synthesis impacted by depolarization point to a central role for the upregulation of mitochondrial proteins and metabolic activity (Figs. 3f,j, 5b and 6) in response to dendritic activation. In agreement with our work, Li et al.^94^ showed that depolarization enhances mitochondrial localization at dendritic spines in a calcium influx-dependent manner.

Taken together, our studies reveal an essential mechanism of activity-dependent uORF:eIF4G2-mediated translational control present in many dendritic transcripts. It remains to be determined if the translated uORFs encode functional micropeptides and how the de novo translation of downstream proteins affects neuronal physiology. This regulation is poised to help maintain neuronal integrity and dendritic function during calcium influx, providing insights into the specialized nature of mechanisms underlying protein synthesis-dependent synaptic plasticity.

## Methods

### Plasmid and reporter cloning

Constructs were cloned into the doxycycline-inducible Lenti-X Tet-ON 3G expression system (Takara Bio, 631187) using the *P*_TRE3GS_ promoter. TurboID was generously provided by A. Y. Ting at Stanford University. PSD95 was amplified from rat PSD95, and the 5′ UTR and 3′ UTR regions of PSD95 were amplified from the pCMV-5U-Venus-PSD-95-3U construct (Addgene, 102949). Superfolder GFP (sfGFP) was included between TurboID and PSD95 to visualize TurboID in live cells, and Flag tag was included at the 5′ end of TurboID to use in immunostaining. In the final 5′ UTR-TurboID-sfGFP-PSD95-3′ UTR construct, TurboID-sfGFP-PSD95 were separated by GS and NSRV linkers, respectively. All viral constructs were cloned and propagated at 30 °C. Control experiments to test the leakiness and doxycycline sensitivity of the Tet-ON 3G system were done using the luciferase reporter that was included in the Takara kit. Luciferase activity was measured using the Varioskan LUX plate reader and the SkanIt RE 5.0 program. All constructs were expressed at 300 ng ml^−1^ doxycycline concentration.

Myristoylation (Met-Gly-Thr-Val-Leu-Ser-Leu-Ser-Pro-Ser-Tyr) and LDLRct sequences were cloned at the 5′ and 3′ end of sfGFP, respectively, using the Gibson strategy (New England Biolabs (NEB), E5510S). Myristoylation, LDLRct, BC1 and Camk2a-3UTR were amplified from neuronal cDNA. 5′ UTRs were either amplified from neuronal cDNA or generated via gene blocks at Integrated DNA Technologies (IDT). All constructs were cloned and propagated at 30 °C. 5′ UTR and uORF mutations and eIF4G2 phosphorylation mutations were performed using a Q5 Site-Directed Mutagenesis Kit (NEB, E0554S). All clones were confirmed with forward and reverse sequencing primers using GENEWIZ Sanger sequencing services.

### Lentivirus preparation and lentiviral transduction

Human embryonic kidney (HEK) 293T cells (American Type Culture Collection, CRL-11268) were cultured in DMEM (Thermo Fisher Scientific, 11965092) supplied with 10% FBS (Thermo Fisher Scientific, SH3007103), 2 mM L-glutamine (Thermo Fisher Scientific, 25030081), 1 mM sodium pyruvate (Thermo Fisher Scientific, 11360070) and 1× non-essential amino acids (Thermo Fisher Scientific, 11140076) at 37 °C under 5% CO_2_. The cells were passaged at 80–90% confluence by trypsinization fewer than 20 times to make the lentivirus and were seeded at 85,000 cells per cm^2^ confluence in 10-cm dishes 1 d before transfection. High-titer lentivirus mixes were prepared using Lenti-X Packaging Single Shots (Takara Bio, 631275) according to the manufacturer’s guidelines. HEK293T cells were incubated with the transfection media for 4–12 h, after which 6 ml of fresh media was added onto the plates. After 48 h, the supernatant was collected carefully from the dishes, centrifuged at 500*g* for 10 min and subsequently filtered through a 0.45-µm syringe filter. Viral soups were either flash frozen with liquid nitrogen and stored at −80 °C or used immediately. Lentivirus aliquots, which were used for the sequencing experiments, were not freeze–thawed more than once. Optimal expression of each fusion construct and the titer were determined experimentally for Pan-TurboID and TurboID-PSD95.

### Primary cortical cultures

Pregnant mice were purchased from Charles River Laboratories (albino, CD-1, strain 022) and treated according to Institutional Animal Care and Use Committee guidelines at The Rockefeller University. Embryonic day (E) 14.5 embryos were sacrificed in 1× HBSS. Dissected embryonic cortical tissues were gently dissociated and digested for 30 min at 37 °C and 5% CO_2_ with a combination of Papain and DNaseI according to the manufacturer’s instructions (Worthington Biochemical, LK003150). Digested tissues were filtered through a 100-μm mesh and centrifuged at 1,000 r.p.m. for 4 min at room temperature. The cells were then seeded and grown in Neurobasal Medium (Thermo Fisher Scientific, 21103049) supplemented with 2% (v/v) B-27 (Thermo Fisher Scientific, 17504044) and 1% (v/v) GlutaMAX (Thermo Fisher Scientific, 35050061) at approximately 25,000 cells per cm^2^ on pre-coated dishes. Dishes were pre-incubated with 0.01% poly-l-ornithine at 37 °C and 5% CO_2_ (or room temperature) overnight, washed three times with sterile distilled water and dried before plating the cells. Additional neurons that were not going to be treated with virus were plated in separate dishes to provide conditioned media for the experimental cells. Virus was added the next day, and the media was replaced with half fresh media and half conditioned media supplied with 300 ng ml^−1^ doxycycline after 24 h. Primary neuronal cells were grown in vitro for 12 d, replacing one-third of the media with half fresh and half conditioned media (with doxycycline) every 3 d.

### Neuronal activation by KCl depolarization

Before KCl depolarization on the 12th day, neurons were silenced with 1 µM sodium channel blocker tetrodotoxin (TTX) (Abcam, ab120054) and 100 µM NMDA receptor antagonist DL-2-amino-5-phosphopentanoic acid (DL-AP5) (Abcam, b120004) for 2 h at 37 °C and 5% CO_2_. TTX was dissolved in pH 4.8 citrate buffer to 1 mM, and DL-AP5 was dissolved in water to 10 mM as stock concentrations. Subsequently, neurons were activated for 1 h by adding warm KCl depolarization buffer (170 mM KCl, 2 mM CaCl_2_, 1 mM MgCl_2_ and 10 mM HEPES in Neurobasal Medium) to a final concentration of 33% of the total culture medium in the plate. Whole medium was then replaced with fresh neurobasal medium for 30 min, including biotin, TTX and DL-AP5 (biotin was included for all of the labeling experiments).

### Neuronal activation by DHPG

Before depolarization on the 12th day, neurons were silenced as described in the ‘Neuronal activation by KCl depolarization’ subsection. After silencing, neurons were washed with regular media twice and incubated in regular media for 15 min before they were activated with 100 µM DHPG ((R.S.)-3,5-dihydroxyphenylglycine) (Tocris, 0805) for 10 min. DHPG medium was then replaced with fresh Neurobasal Medium for 20 min, during which biotinylation was performed.

### Biotin labeling with TurboID in primary mouse cortical neurons

Cells were incubated with 100 μg ml^−1^ cycloheximide (Chx) for 2 min before adding 100 μM biotin for 30 min at 37 °C and 5% CO_2_. Inducible and controlled expression of TurboID was critical for site-specific biotinylation in neurons, consistent with the observation that constitutive expression of TurboID leads to promiscuous biotin ligase activity^21^. For the minus biotin samples, cells were incubated with Chx only for the same amount of time as the plus biotin samples, and all were harvested at the same time. For activated neurons, biotinylation was induced after replacing the KCl media with fresh media, which included TTX and DL-AP5 that were used to silence neurons before depolarization.

### Reporter transfection

After 10 d in culture, primary cortical neurons were transfected using Lipofectamine LTX with PLUS reagent (Thermo Fisher Scientific, 15338030). For one well of a 12-well plate, TET (0.6 µg) was co-transfected along with the reporter constructs (0.6 µg) using 1.6 µl of LTX and 1.2 µl of PLUS reagents. Media was changed with fresh media including doxycycline (300 ng ml^−1^) after 4–6 h, and cells were harvested within 12–14 h.

### Fura-2 AM and Fluo-4 AM staining and fluorescence measurement

Fura-2 AM (Abcam, ab120873) stock solution was prepared in DMSO at 10 mM. Resting cells were loaded with the Fura-2 AM dye simultaneously with the depolarized cells at a final concentration of 2 µM. The dye was added along with the KCl solution to the depolarized cells, and the cells were incubated for 45 min at 37 °C and 5% CO_2_. Then, cells were washed with neurobasal media three times and were kept in neurobasal media without Fura-2 AM at 37 °C and 5% CO_2_ for another 30 min. The fluorescence was then imaged using a Keyence microscope at the excitation wavelength 340/380. Fluorescent nuclei were counted using ImageJ software.

Fluo-4 AM (Thermo Fisher Scientific, F14201) was resuspended in DMSO to 1 mM. Similar to the Fura-2 AM strategy, resting and depolarizing cells were loaded with the Fluo-4 AM at 2 µM simultaneously at the beginning of depolarization for 45 min at 37 °C and 5% CO_2_. Pluronic F-127 (Thermo Fisher Scientific, P6866) was added at 0.02% to help disperse the dye in the media. Cells were then washed with regular neurobasal media three times and were kept in neurobasal media for another 30 min at 37 °C and 5% CO_2_. The fluorescence was then imaged using the Keyence microscope at the excitation wavelength 494/506. Fluorescent nuclei were counted using ImageJ.

In addition to imaging, fluorescence by Fura-2 AM or Fluo-4 AM was measured using the Varioskan LUX plate reader and the SkanIt RE 5.0 program at excitation/emission at 340/380 nm and 494/506 nm, respectively. Each biological replicate was calculated as the average of three wells (technical replicates) in the 96-well plate plated from the same batch of neurons.

### PL-CLIP lysate preparation

For the UV-crosslinking experiments, neurons were washed twice with 1× PBS supplemented with 100 μg ml^−1^ Chx and crosslinked on 150-mm plates on ice in 1× PBS with Chx with one pulse of 400 mJ cm^−^^2^ and one pulse of 200 mJ cm^−^^2^. As described previously by Kaewsapsak et al.^95^ and Hendrickson et al.^96^, cells were lysed in 0.5 ml of ice-cold RIPA buffer (50 mM Tris (pH 8), 150 mM KCl, 0.1% SDS, 1% Triton X-100, 5 mM EDTA, 0.5% sodium deoxycholate, freshly supplemented with 0.5 mM dithiothreitol (DTT), 1× EDTA-free protease inhibitor cocktail (Thermo Fisher Scientific, 87785) and 100 U ml^−1^ RNaseOUT (Life Technologies, 10-777–019)) for 10 min on ice and clarified by centrifugation at 15,000*g* for 10 min at 4 °C. Samples were diluted by adding 0.5 ml of Native Lysis Buffer (NLB) (150 mM KCl, 25 mM Tris pH 7.5, 5 mM EDTA, 0.5% NP-40, 0.5 mM DTT, 1× protease inhibitor and 100 U ml^−1^ RNaseOUT). Lysates were subsequently flash frozen or used for streptavidin pulldowns.

### PL-Ribo-seq lysate preparation and monosome fractionation

For the ORF-RATER experiments, three 150-mm cell culture dishes were combined per sample per replicate. Neurons were treated with nothing (WT), Chx (100 μg ml^−1^) for 2 min or harringtonine (Harr) (2 μg ml^−1^) for 2 min, followed by a Chx pulse. For PL-Ribo-seq experiments, four 150-mm cell culture dishes were combined per sample per replicate, one-fourth of which was spared for proteomics. The PL-Ribo-seq samples were treated with Chx (100 μg ml^−1^) for 2 min, followed by a 30-min biotin pulse. Both for the ORF-RATER and PL-Ribo-seq experiments, cells were quickly rinsed in ice-cold polysome gradient buffer (20 mM Tris pH 7.5, 150 mM NaCl, 5 mM MgCl_2_, freshly supplemented with 1 mM DTT and 100 μg ml^−1^ Chx) and then scraped and lysed on plates on ice in 1 ml of ice-cold polysome lysis buffer (20 mM Tris pH 7.5, 150 mM NaCl, 5 mM MgCl_2_, 1% Triton X-100 (Thermo Fisher Scientific, 85111), freshly supplemented with 20 U ml^−1^ SUPERase-In RNase inhibitor (Thermo Fisher Scientific, AM2694), 24 U ml^−1^ Turbo DNase (Thermo Fisher Scientific, AM2239), 1 mM DTT, 100 μg ml^−1^ Chx and 1× EDTA-free protease inhibitor (MilliporeSigma, 11836170001)). Lysates were clarified by centrifugation at 20,000*g* for 2 min at 4 °C, after which the supernatant was immediately loaded onto the cold 5-ml 7-kDa molecular weight cutoff (MWCO) Zeba desalting column (Thermo Fisher Scientific, 89892) that was previously equilibrated with the ice-cold polysome gradient buffer. This step was tested to be necessary to minimize post-lysis biotinylation and was omitted for the ORF-RATER samples. Lysates were subsequently flash frozen or used for monosome fractionation. For monosome fractionation, lysates were added 5 mM CaCl_2_, incubated with micrococcal nuclease (3 U μg^−1^ of RNA) for 45 min at room temperature, quenched with 6.25 mM EGTA and loaded on sucrose gradients as previously described^97^. Samples were then centrifuged for 2 h at 41,000 r.p.m. in an SW-41 rotor (Beckman Coulter), and monosomes were collected using BioComp Gilson fraction collection (Gilson FC203B collector) and Triax software (BioComp Instruments).

### PL-MS lysate preparation

One-fourth of the lysate from the ribosome profiling experiments before spin was spared for the MS experiments. To this fraction of lysate, SDS and sodium deoxycholate were added at final concentrations of 0.1% and 0.5%, respectively, to increase lysis efficiency and release of membrane proteins. The additional detergent-included lysate was then incubated on ice for 10 min and clarified by centrifugation at 15,000*g* for 5 min at 4 °C. The lysate was then desalted with the Zeba desalting column and either flash frozen or subsequently used for streptavidin pulldowns. Minus biotin samples were prepared the same way but without a biotin incubation step before harvesting the cells.

### Streptavidin pulldowns

For TurboID-CLIP RNA sequencing experiments, 15% of the lysate was taken for input, and 50 µl of RIPA:NLB equilibrated C1 magnetic beads (Thermo Fisher Scientific, 65-001) was added to the remaining lysate. The pulldown was allowed to proceed overnight at 4 °C. Beads were then washed briefly at 4 °C with ice-cold (1) RIPA buffer twice; (2) high-salt buffer (1 M KCl, 50 mM Tris, pH 8, 5 mM EDTA); (3) urea buffer (2 M urea, 50 mM Tris, pH 8, 5 mM EDTA); (4) RIPA buffer; (5) 1:1 RIPA:NLB; (6) NLB; and (7) TE (10 mM Tris, pH 7.5, 1 mM EDTA). All buffers were supplemented with 100 U ml^−1^ RNaseOU. The inputs and beads were then treated with sarkosyl and proteinase K (2% N-lauryl sarkosyl, 10 mM EDTA, 5 mM DTT, in 1× PBS, supplemented with 200 µg of proteinase K (Roche) and 4 U of RNaseOUT) at 42 °C for 1 h, followed by 55 °C for 1 h to digest the biotinylated proteins and free the bound RNAs. RNA isolation was then performed using TRIzol (Thermo Fisher Scientific, 15596026) according to the manufacturer’s instructions.

For the ribosome profiling experiments, 10% of the monosome fraction was kept for input. Triton X-100 was added to the remaining monosomes to a final concentration of 0.05%. The biotinylated monosomes were isolated using 50 µl of MyOne streptavidin C1 magnetic Dynabeads that were prepared according to the manufacturer’s guidelines and washed twice with Buffer A (100 mM NaOH, 50 mM NaCl), twice with Buffer B (100 mM NaCl) and once with polysome gradient buffer (with DTT and Chx). The monosome and bead mix was mutated at 4 °C overnight. The supernatant was removed the next day, and the beads were moved into a new tube with low-salt wash buffer (20 mM Tris pH 8, 150 mM NaCl, 5 mM MgCl_2_, 1 mM DTT, 100 μg ml^−1^ Chx and 0.1% Triton X-100) and consequently washed three times for 10 min at 4 °C with high-salt wash buffer (20 mM Tris pH 8, 500 mM NaCl, 5 mM MgCl_2_, 1 mM DTT, 100 μg ml^−1^ Chx and 0.1% Triton X-100). The beads were then moved into a new tube with the low-salt binding buffer, and RNA extraction was performed on the pulldowns and inputs using TRIzol.

Pulldowns for the MS and control pulldown experiments were performed on whole cell lysates described in the ‘PL-MS lysate preparation’ subsection. The lysates were diluted five-fold with 1× PBS. Then, 50 µl of C1 magnetic Dynabeads was washed three times with 1× PBS and incubated with the lysates for 30 min at room temperature, followed by at 4 °C overnight. The next day, beads were moved into a new tube with the low-salt wash buffer and washed with the high-salt wash buffer twice for 5 min at 4 °C. After the high-salt washes, beads were washed once with 0.5% Tween 20 (in 10 mM Tris pH 8) for 5 min at 4 °C, once with 2 M urea (in 10 mM Tris pH 8) for 3 min at room temperature and three times with 10 mM Tris pH 8 for 3 min each at room temperature, changing tubes each time for the last three washes. Changing the tubes notably reduced the detergent contamination in the samples.

### Library preparation

For PL-CLIP, total RNA (input and pulldown, ~100 ng) was used as the starting material and first treated with DNAse on columns. TruSeq stranded RNA sample protocol was followed using RiboZero to get rid of ribosomal RNAs (Illumina, 20020594). For unique adapters, IDT Illumina TruSeq RNA UD Indexes (Illumina, 20022371) were used. The samples were then pooled and sequenced on an Illumina NovaSeq 6000 sequencer.

For ribosome profiling libraries, input and pulldown samples were prepared with barcoded linkers as described previously^97,98^. Pooled libraries were then sequenced on the Illumina NovaSeq 6000 sequencer.

### MS

Proteins coupled to streptavidin-coated magnetic beads were reduced with 10 mM DTT and alkylated with 25 mM iodoacetamide (Sigma-Aldrich). Proteins were eluted from beads using partial on-bead trypsinization with 0.5 µg of trypsin (Promega), followed by a second digestion with a 1:1 solution of 0.5 µg of trypsin (Promega) and 0.5 µg of LysC (Wako). Samples were then solid-phase extracted using C18 micro-purification tips constructed in-house and analyzed by liquid chromatography with tandem mass spectrometry (LC–MS/MS). For LC–MS/MS, samples were separated by reversed-phase chromatography using an analytical gradient (98% A, 2% B and 62% A, 38% B where A is 0.1% formic acid and B is 80% acetonitrile in 0.1% formic acid) for 50 min in 12-cm built-in-emitter columns. Spectra were collected using an Orbitrap Fusion LUMOS (Thermo Fisher Scientific). Then, spectra were queried against a Mouse UniProt proteome FASTA database (March 2020) using Proteome Discoverer 1.4 (Thermo Fisher Scientific) and Mascot 2.4 (Matrix Science). Perseus version 1.6.10.50 was used for data analysis. Matched proteins were filtered for possible contaminants. Proteome Discoverer 1.4–calculated protein abundances (average of the three most abundant peptides for a matched protein) were log_2_ transformed. Matched proteins were filtered, keeping only proteins with signals in a minimum two-thirds of the replicates for at least one condition. Missing signals were imputed (width, 0.3; downshift, 1.8), followed by a quartile-based width adjustment normalization as described in the Perseus software documentation. Overall, 4,418 proteins were detected, and 2,776 unique ones that passed all the quality controls were used for the downstream analyses. Resting and depolarized enriched proteomes were determined with significance cutoff of log_2_ > 0 and *P* < 0.05 using the two-tailed, paired Student’s *t*-test on Pan-TurboID versus TurboID-PSD95 replicates after the minus biotin counterpart was subtracted from each sample. Differential (depolarized minus resting) enriched proteome was determined with significance cutoff of log_2_ > 0 and *P* < 0.05 using the two-tailed, unpaired Student’s *t*-test on resting versus depolarized replicates (Supplementary Table 2).

### IF

In brief, neurons were grown in two-well or four-well chambered Nunc Lab-Tek II coverglasses that were previously coated with poly-l-ornithine as described in the ‘Primary cortical cultures’ subsection. Cells were rinsed with 1× PBS twice and fixed with 4% paraformaldehyde (PFA) at room temperature for 10 min. After fixation, cells were washed with 1× PBS three times and permeabilized in 0.2% Triton X-100 (in 1× PBS) for 10–15 min on ice. Cells were washed again three times with 1× PBS and blocked in 3% donkey or goat serum (depending on the secondary antibody) diluted in 1× PBS for 1 h at room temperature. Primary antibody diluted in 3% serum in 1× PBS was added for overnight incubation at 4 °C, followed by three 1× PBS washes the next day and secondary antibody (diluted in 3% serum in 1× PBS) incubation for 2 h at room temperature. Three 1× PBS washes were performed after the secondary antibody incubation, and DAPI was added during the second wash. The cells were kept in 1× PBS at 4 °C in dark until imaging. A Keyence Bz-9000e fluorescence microscope was used to image IF samples. IF images were quantified in ImageJ using the same threshold parameters across resting and depolarized conditions. To calculate the dendritic signal specifically, MAP2 co-localization was used as a proxy, and the signal surrounding the soma was subtracted. ROI Manager was used to select dendrites and measure fluorescence intensity, and background was subtracted for each image.

### RNA FISH

RNA FISH was performed using the RNAscope assay according to the manufacturer’s instructions. In brief, for primary cortical neurons, fixation was performed with 4% PFA for 30 min at room temperature. The cells were then washed with 1× PBS twice and dehydrated with 50% EtOH, 70% EtOH and 100% EtOH with each incubation for 5 min, performed twice. The cells were left in 100% EtOH at −20 °C at least for half an hour before proceeding with the rest of the FISH protocol. Before protease treatment, the cells were rehydrated with 70% EtOH and 50% EtOH for 2 min at room temperature and washed with 1× PBS. ProteaseIII from the RNAscope kit was diluted 15-fold in RNAse-free water, and the diluted protease was applied to cells for 10 min at room temperature. The rest of the incubations for the ACD probe (pTHSSe-sfGFP-C2, ACD Bio, 844781-C2) and washes were performed according to the manufacturer’s instructions. When combining FISH with IF, one of the channels was spared for IF, and the amplification step for that channel was omitted. Instead, cells were blocked with 3% serum in 1× PBS for 1 h at room temperrature and then incubated with the primary antibody in 3% serum overnight at 4 °C. The rest of the IF protocol was followed as described in the ‘IF’ subsection. Imaging slides were kept in ProLong Gold Antifade (Thermo Fisher Scientific, P10144) with a coverslip at 4 °C for a few weeks or at −20 °C for longer periods. Samples were imaged using the Keyence Bz-9000e fluorescence microscope. FISH signals were quantified using the ImageJ ‘threshold’ and ‘analyze particles’ features. Signals within DAPI and in MAP2 were counted separately to differentiate the soma and dendrites.

### PLA

PLAs were performed to detect nascent protein production in neurons. To detect new protein production, puromycin (Thermo Fisher Scientific, A1113803) was incorporated at 2 µM for 10 min at 37 °C and 5% CO_2_. Cells were then washed with pre-warmed PBS-MC (1× PBS pH 7.4, 1 mM MgCl_2_, 0.1 mM CaCl_2_), fixed in PBS-MC supplemented with 4% PFA and 4% sucrose, washed with PBS-MC again and permeabilized in 0.5 % Triton X-100 in 1× PBS for 15 min as described previously^99^. Anti-puromycin and protein-specific antibodies were used in combination for each protein of interest. Blocking, PLA probe application, ligation and amplifications were performed using the Duolink kit (Sigma-Aldrich, DUO92101) according to the manufacturer’s recommendations. Samples were imaged and quantified as described in the ‘RNA FISH’ subsection.

### Western blots

Lysates were boiled at 95 °C for 5 min with NuPAGE LDS Sample Buffer (NP0007) and NuPAGE Sample Reducing Agent (NP0009) and run in 4–12% Bis-Tris gels. Protein transfers were performed using the iBlot2 nitrocellulose dry transfer system (at 20 V for 6 min for proteins <20 kDa and 25 V for 7 min for >20 kDa). The membranes were blocked in Intercept TBS Blocking Buffer for 1 h at room temperature (LI-COR Biosciences, 927-60001). TBS buffer performed better in minimizing the background signal, especially for streptavidin blots. Primary antibodies were diluted according to their specifications in the Intercept buffer and incubated with the membranes on orbital shakers overnight at 4 °C. The next day, membranes were washed in TBST (1× TBS with 0.1% Tween 20) for 5 min at room temperature three times and incubated with the secondary antibodies diluted in TBST for 2 h at room temperature. If the membranes were going to be blotted for biotin, they were incubated with the streptavidin antibody diluted in TBST for 7–10 min at room temperature after the secondary antibody incubation. The membranes were washed again in TBST for 5 min at room temperature three times. Two more PBS washes were added if the membranes were blotted for streptavidin.

The samples for reporter constructs were divided into two, one half for the western and the other for RNA extraction for each biological replicate. If sequential antibody incubation was performed, the western blots were stripped at room temperature or at 37 °C (if high-affinity antibody was used) using the Restore PLUS Stripping Buffer (Thermo Fisher Scientific, 46430) for 10–15 min and subsequently blocked in the Intercept TBS Blocking Buffer before the next antibody incubation.

### Quantitative polymerase chain reaction

Total RNA was prepared using TRIzol after the lysates were treated with RQ1 DNase (Promega, M6101) according to the manufacturer’s instructions. cDNA was generated using iScript reverse transcription mix (Bio-Rad, 1708891), and quantitative polymerase chain reaction (qPCR) was performed using the FastStart SYBR Green Master (Roche, 04673492001). The forward and reverse primers used were: TACCGTTAGCCCCTATGCCATC and CTCGGTTGCCCATCCTCACC for *Arc*; ATGCTCCCCGGGCTGTATTC and GATCTTCTCCATGTCGTCCCAG for *β-Actin*; AACACACAGGACTTTTGCGC and GCTCTGGTCTGCGATGGG for *Fos*; ATGACTGCAAAGATGGAAACG and CAGGTTCAAGGTCATGCTCT for *Jun*; CAGACAACCATTACCTGTCGAC and CTCTGTGGTCTTCTGGTAGACT for the GFP reporter; GCACTAAGCCGAATGCCTTCT and CTCTGTGGTCTTCTGGTAGACT for the *Mphosph9* reporter; and CTCGATGCCCATCTCTACTGGT and CTCTGTGGTCTTCTGGTAGACT for the *Kcnj9* reporter.

### Antibodies and fluorescent dyes

#### Fluorescent streptavidin conjugate

Streptavidin (1:5,000 for western blots and 1:10,000 for IF; Thermo Fisher Scientific, S32358).

#### Primary antibodies

Puromycin (1:3,000, mouse, Kerafast, EQ0001, RRID: AB_2620162), Flag (1:3,000, mouse, Sigma-Aldrich, F1804, RRID: AB_262044), β-Actin antibody (1:2,500, mouse, Sigma-Aldrich, A1978, RRID: AB_476692), RPL10A (1:1,000, rabbit, Abcam, ab174318), MAP2 (1:2,500, guinea pig, Synaptic Systems, 188004, RRID: AB_2138181), GFAP (1:500, rabbit, Abcam, ab7260, RRID: AB_305808), OLIG2 (1:500, rabbit, Proteintech, 13999-1-AP, RRID: AB_2157541), PSD95 (1:500, mouse, Millipore, MABN68, RRID: AB_10807979), Synaptophysin (1:300, mouse, Abcam, ab8049, RRID: AB_2198854), SHANK3 (1:500, mouse, Novus, NBP1-47610, RRID: AB_10010567), GKAP (1:500, rabbit, Novus, NBP1-76911, RRID: AB_11017331), NLGN1 (1:200, mouse, Novus, NBP2-42192), HOMER1 (1:1,000, rabbit, Proteintech, 12433-1-AP, RRID: AB_2295573), GAPDH (1:5,000, mouse, Thermo Fisher Scientific, AM4300, RRID: AB_2536381), BAIAP2 (1:500, rabbit, Proteintech, 11087-2-AP, RRID: AB_2063075), DLGAP3 (1:500, rabbit, Proteintech, 55056-1-AP, RRID: AB_10858793), TBR1 (1:500, rabbit, Proteintech, 20932-1-AP, RRID: AB_10695502), H4 (1:1,000, mouse, Abcam, ab31830, RRID: AB_1209246), H2A.X (1:1,000, rabbit, Proteintech, 10856-1-AP, RRID: AB_2114985), EEF2 (1:1,000, rabbit, Cell Signaling Technology, 2332, RRID:AB_10693546), P-EEF2 (1:1,000, rabbit, Cell Signaling Technology, 2331, RRID: AB_10015204), eIF2α (1:1,000, rabbit, Cell Signaling Technology, 9722, RRID: AB_2230924), P-eIF2α (1:1,000, rabbit, Cell Signaling Technology, 3398, RRID: AB_2096481), p42/44 MAPK (1:1,000, rabbit, Cell Signaling Technology, 4695, RRID: AB_390779), P-p42/44 MAPK (1:1,000, rabbit, Cell Signaling Technology, 9101, RRID: AB_331646), P-IRE1 (1:500, rabbit, Novus, NB100-2323SS, RRID: AB_10145203), IRE1 (1:500, rabbit, Novus, NB100-2324SS, RRID: AB_10000972), CHOP (1:1,000, mouse, Cell Signaling, 2895T, RRID: AB_2089254), ATF4 (1:1,000, rabbit, Cell Signaling Technology, 11815S, RRID: AB_2616025), MPHOSPH (1:300, rabbit, Biorbyt, orb100446), KCNJ9 (1:300, rabbit, LSBio, LS-C352416), KCNJ9 (1:300, mouse, Antibodies Incorporated, 75-445, RRID: AB_2686912), eIF4G2 (1:1,000, rabbit, Cell Signaling Technology, RRID: AB_10622189 and rabbit, Cell Signaling Technology, RRID: AB_2261993), NSUN3 (1:250, rabbit, LSBio, LS-C163024), MTF1 (1:300, rabbit, Novus, NBP1-86380, RRID: AB_11011361), ZFP64 (1:300, rabbit, Proteintech, 17187-1-AP, RRID: AB_2218826) and KATNBL1 (1:250, rabbit, Proteintech, 24795-1-AP, RRID: AB_2879730).

#### Secondary antibodies

The dilution used for the secondary antibodies was 1:1,000. Donkey anti-rabbit 800 (LI-COR Biosciences, 926-32213), donkey anti-rabbit 680 (LI-COR Biosciences, 926-68073) and goat anti-mouse 800 (LI-COR, 926-32210) for western blots. Donkey anti-mouse Alexa Fluor 488 (Thermo Fisher Scientific, R37114, RRID: AB_2556542), donkey anti-guinea pig Alexa Fluor 488 (Jackson ImmunoResearch, 706-545-148, RRID: AB_2340472), donkey anti-rabbit Alexa Fluor 647 (Jackson ImmunoResearch, 711-605-152, RRID: AB_2492288) and donkey anti-mouse Alexa Fluor 555 (Thermo Fisher Scientific, A-31570, RRID: AB_2536180).

### siRNA knockdowns

Knockdown of eIF4G2 was performed using Lipofectamine LTX with PLUS reagent (Thermo Fisher Scientific, 15338030) at greater than 85% confluency at days 8–9 in vitro for 48–72 h. The siRNA for eIF4G2 (Santa Cruz Biotechnology, sc-35170) was optimal at 20 nM final. A non-targeting siRNA control was included for both resting and depolarized conditions.

For the eIF4G2 rescue experiments, knockdown of eIF4G2 was performed using 15 nM siRNA and 1.4 µl of LTX and 1 µl of PLUS reagents to keep the neurons healthier for the next round of transfections, and neurons were transfected with the dendritic eIF4G2 variants 48 h after siRNA knockdown as described in the ‘Reporter transfection’ subsection. The eIF4G2 variants were conjugated to myristoylation and LDLR-C-terminal sequences to localize them in dendrites (similar to our dendritic reporters), which rendered the size of eIF4G2 to be higher and allowed us to see the levels of endogenous eIF4G2 knockdown (Extended Data Fig. 10d).

### Calcium deprivation by EGTA

Resting or silenced neurons were treated with 10 mM EGTA for 10 min at 37 °C and 5% CO_2_ to test if the KCl-mediated effects on translation were dependent on calcium influx. For the depolarized cells, KCl was added after the EGTA treatment.

### Induction of stress

Neurons were treated with 0.5 mM sodium arsenite (NaAsO_2_) (Sigma-Aldrich, S7400) at 37 °C and 5% CO_2_ for 1.5 h.

### CLIP

Neurons were washed twice with 1× PBS with 100 μg ml^−1^ Chx and UV crosslinked on ice in the same wash buffer with one pulse of 400 mJ cm^−^^2^ and one pulse of 200 mJ cm^−^^2^. Cells were then immediately scraped in fresh 1× ice-cold PBS with Chx and centrifuged at 5,300*g* for 5 min at 4 °C. The pellets were flash frozen or processed as described previously^14,100^ with modifications. Five biological replicates were used, each replicate prepared from three 15-cm cell culture dishes. Pellets were resuspended in 0.5 ml of lysis buffer (1× PBS, 0.1% SDS, 0.5% NaDOC, 0.5% NP-40 with freshly added protease inhibitor), and immunoprecipitations were performed overnight at 4 °C using the rabbit monoclonal antibody against eIF4G2. Resting and depolarized samples were pooled after barcoding at the reverse transcription step to increase yield.

### Bioinformatics

#### PL-CLIP analysis

Transcript expression was quantified from RNA sequencing (RNA-seq) reads using salmon and mm10 UCSC knownGene gene models using the ‘TxDb.Mmusculus.UCSC.mm10.knownGene’ Bioconductor package. The longest transcript for each gene was used for all analyses. Lowly expressed genes were filtered with edgeR’s ‘filterByExpr’ command^101^. limma’s ‘voomWithQualityWeights’^102,103^ was used for differential gene expression analysis by grouping pulldown samples according to condition (rest versus dep) and bait (Pan versus PSD95). To determine transcripts that are localized in a given condition, limma was used to compare the TurboID-PSD95 pulldown to the Pan-TurboID pulldown (PSD95 − Pan). For depolarized versus resting comparisons, these contrasts were compared ((Dep-PSD95 − Dep-Pan) − (Rest-PSD95 − Rest-Pan)). The design model included group and batch (for the four replicates). Multiple test correction was performed using the ‘decideTests’ function, using the Benjamini–Hochberg method. Localized transcripts were defined by *t*-statistic >1 in resting and depolarized and >1.25 for depolarized versus resting comparisons (Supplementary Table 1).

#### PCA

PCA was calculated by taking the top 500 variable genes into account and using the ‘prcomp’ and ‘pcascree’ functions in RStudio. For visualization, the ‘fviz_pca_ind’ function from the ‘factoextra’ package was used.

#### GSEA and GSEA-based analysis to compare RNA-seq, Ribo-seq and proteomics datasets

To identify the gene sets enriched in the relevant dendritic datasets, GSEA was performed using the ‘fgsea’ package from Bioconductor (https://www.biorxiv.org/content/10.1101/060012v3), using false discovery rate (FDR) < 0.05 and default settings unless otherwise stated.

For the dataset comparisons, instead of using pathways (Gene Ontology terms) in the ‘fgsea’ package, actual gene names from the list that was being compared were fed to the ‘fgsea’ function. For Fig. 2a and Extended Data Figs. 2c and 4e, genes were ranked according to dendritic enrichment in resting PL-CLIP; for Extended Data Fig. 5d,h, genes were ranked by dendritic enrichment in resting PL-Ribo-seq; for Extended Data Fig. 5j, genes were ranked by dendritic enrichment in differential PL-Ribo-seq; and for Extended Data Fig. 4b,c, genes were ranked by dendritic enrichment in resting PL-MS.

#### PL-Ribo-seq data analysis

FASTQ files were processed using the Bioconductor ‘Rfastp’ package. Adapters were trimmed, and low-quality reads were removed using the same package. Demultiplexing was performed using ‘fastq-multx’. Six-nucleotide unique molecular identifiers were removed from the 5′ end. Finally, reads longer than 20 nucleotides were used for the following analyses. The processed FASTQ files were mapped to mm10 (UCSC) with ‘Rsubread’^104^. The counts of each transcript in each sample were calculated by Plastid^53,58^ with the annotation GTF file generated from the ‘TxDb.Mmusculus.UCSC.mm10.knownGene’ package of Bioconductor. Raw counts and reads per kilobase per million mapped reads (RPKM) per transcript were generated, and the longest transcript per gene was selected. The RPKMs of each sample were merged and normalized with quantile normalization.

riboWaltz^52^ was applied to assess ribosome profiling quality control. In brief, the transcript-aligned BAM files were generated by STAR. Then, the quality control plots, including trinucleotide periodicity and P-site phasing, were generated according to the instructions in riboWaltz.

CDS dendritic translation for each sample was determined by normalizing TurboID-PSD95 pulldown to the average of Pan-TurboID and TurboID-PSD95 inputs. Dendritic translation in resting and depolarized neurons was calculated by taking the log_2_ transforms of normalized RPKM values of CDSs in each condition. To test the significance of dendritic translation in resting and depolarized data, we used the likelihood ratio test (LRT) in edgeR. In brief, the counting matrix of transcripts in each replicate was modeled with a genewise negative binomial generalized linear model. Then, dendritic translation and *P* values were tested with the LRT with basemean >1. Differential CDS dendritic translation (depolarized minus resting) was calculated by taking the difference of the depolarized and resting log_2_ RPKM of TurboID-PSD95 pulldown and average of TurboID-PSD95 and Pan-TurboID inputs for the longest transcript per gene. To test the significance of differential expression, the log_2_ fold change of each transcript was *z-*transformed. The *P* value of each transcript was calculated based on the normal distribution of z-score (cutoff: ±1.96, 2.5% top/bottom). If the basemean value for a transcript was <1 in the differential but >1 in the corresponding resting and depolarized sets, then the value of the highest expressed transcript was used for the differential. For each transcript, the normalized counts for each replicate were calculated. For significance cutoff, log_2_ fold change >0 with *P* < 0.05 was used unless otherwise stated (Supplementary Table 3).

To determine the ribosome occupancy in the 5′ UTRs, RPKMs, segregated by Ensembl transcript ID, of 5′ UTRs in each sample were calculated with Plastid^53,58^. Then, the RPKM changes for each region between resting and depolarized conditions were tested using the permutation *t*-test. The *P* values were then adjusted with Bonferroni correction.

5′ UTR dendritic translation in resting and depolarized ribosome profiling data was calculated by taking the difference of the log_2_ RPKM of TurboID-PSD95 pulldown and average of TurboID-PSD95 and Pan-TurboID inputs for the longest transcript per gene with basemean cutoff of 3. Translation of all the detected uORFs was calculated by taking into account transcript levels using PL-CLIP data to establish that the observed effects are independent of RNA level changes in dendrites (*t*-stat for both conditions <1 out of all the detected transcripts) and mediated by translational control (Fig. 3h). To establish more stringent, dendritically enriched translated 5′ UTRs, the difference between the log_2_ RPKM of TurboID-PSD95 and Pan-TurboID enrichments was considered for the longest transcript per gene in the differential data (increased with depolarization, depolarized minus resting). If the basemean value for a transcript was <1 in the differential but >1 in the corresponding resting and depolarized sets, then the value of the highest expressed transcript was used for the differential. To determine the significance, the log_2_ fold change of each transcript was *z-*transformed. The *P* value of each transcript was calculated based on the normal distribution of z-score (cutoff: ±1.96, 2.5% top/bottom) (Supplementary Table 5).

Dendritic targets with increased 5′ UTR and CDS translation were determined by >0 of log_2_ and *P* < 0.05 cutoff, taking into account transcript levels determined by TurboID-PSD95 RNA-seq data (differential PL-CLIP *t*-stat <2). Dendritic targets with increased 5′ UTR but decreased CDS translation were determined as >0 of log_2_ and *P* < 0.05 cutoff, taking into account transcript levels determined by TurboID-PSD95 RNA-seq data (differential PL-CLIP *t*-stat >−2) (Supplementary Table 5).

#### RBP motif analysis

Motifs (in the form of position weight matrices) for RBPs were obtained from RBPmap (http://rbpmap.technion.ac.il/) for mouse^72^. A background list was made based on all expressed transcripts in primary cortical neurons determined by our PL-CLIP data using the longest transcript per gene. Motif search was performed on 5′ UTR sequences for transcripts of interest using the ‘countPWM’ function in the ‘Biostrings’ package using a minimum score of 95%. Hypergeometric testing was performed to test for enrichment of motifs among dendritically translated 5′ UTRs (when compared to all expressed 5′ UTRs) with FDR < 0.1. The ‘seqLogo’ and ‘pheatmap’ packages were used to visualize the binding motifs and generate the heatmaps, respectively.

RBP sites in 5′ UTRs were determined by defining the dendritically increased 5′ UTR translation as >0 of log_2_, *P* < 0.05 in 5′ UTR PL-Ribo-seq and dendritically increased CDS as >0 of log_2_, *P* < 0.05 and dendritically decreased CDS as <0 of log_2_, *P* < 0.05 (Fig. 5a) in CDS PL-Ribo-seq, taking into account transcript levels determined by TurboID-PSD95 RNA-seq data (differential PL-CLIP *t*-stat <2 for increased and *t*-stat >−2 for decreased CDS targets). For comparison of RBP binding sites in dendritic 5′ UTRs enriched in resting, depolarized and differential (depolarized minus resting) PL-Ribo-seq (Extended Data Fig. 7a), the top approximtely 800 significant genes in each category were considered based on the basemean cutoffs mentioned in the ‘PL-Ribo-seq data analysis’ section (5′ UTR *P* < 0.01). For this analysis, transcript levels were taken into consideration, adjusting the lists according to PL-CLIP for each condition (resting and depolarized: *t*-stat <1 and differential: *t*-stat <1.3).

#### ORF-RATER analysis

For processing of ribosome profiling data, linker sequences were removed from sequencing reads, and samples were de-multiplexed using FASTX-clipper and FASTX-barcode splitter (FASTX-Toolkit). Unique molecular identifiers and sample barcodes were then removed from reads using a custom Python script. Bowtie version 1.1.2 was used to filter out reads aligning to rRNAs and contaminants, and all surviving reads were aligned to the mouse transcriptome with TopHat version 2.1.1 using –b2-very-sensitive–transcriptome-only–no-novel-juncs–max-multihits = 64 flags. These alignments were assigned a specific P-site nucleotide using a 12-nucleotide offset from the 3′ end of reads. The ORF-RATER pipeline (https://github.com/alexfields/ORF-RATER) was run starting with the BAM files as previously described^58,105^. All uORFs longer than nine nucleotides including the stop codon were considered with orfrater score of >0.7. For uORF comparisons with eIF4G2 CLIP and PL-Ribo-seq datasets, orfrater score >0.6 was used.

#### CLIP analysis

CLIP libraries were sequenced on an Illumina MiSeq to obtain 75-nucleotide, single-end reads. CLIP reads were processed as described previously^106,107^ using CLIP Tool Kit software to filter for quality, demultiplex, remove 5′ and 3′ linker sequences and collapse exact duplicates. The resulting reads were mapped to the mm10 genome using the ‘align’ function from the ‘Rsubread’ package^104^, allowing a maximum of five mismatches and a minimum fragment length of 20. 5′ UTR sequences were extracted from the ‘TxDb.Mmusculus.UCSC.mm10.knownGene’ R package, and ‘summarizeOverlaps’ from the ‘GenomicAlignments’ package^108^ was used to count eIF4G2-CLIP reads over each 5′ UTR. The longest transcript was used for each gene. The number of reads mapping to each 5′ UTR was normalized for library depth, and log_2_ (fold change, depolarized versus resting) values were calculated using a pseudocount of 0.1. Binomial tests were performed using the normalized CLIP tag values (Supplementary Table 6). To test the dendritic translation and localization of eIF4G2-bound targets in response to depolarization, log_2_ fold change >0 and *P* < 0.2 were used for eIF4G2 CLIP. To intersect eIF4G2-bound targets with the set of RNAs with enhanced 5′ UTR or CDS translation in dendrites, fold change >1 was used for the eIF4G2 CLIP and PL-Ribo-seq (with basemean >1 and differential PL-CLIP *t*-stat <2) lists.

### Statistics and reproducibility

Representative images in Figs. 1b,c,e, 3a,b and 4a,b and Extended Data Figs. 1a,c–e,g and 6d,e,g,i were replicated independently at least three times. Biological replicates were processed (for reporter western blots, imaging and qPCRs) by E.H. and N.N. independently and were replicated independently. The number of biological replicates for each corresponding experiment is reported in the figure legends. No data exclusion was performed.

No sample size calculation was performed, but extensive work in sequencing and MS, particularly performed in neurons, informed our choices of minimal number of sample sizes to provide the required statistical power^1,13,14,100^. We increased the number of biological replicates to four and five for our sequencing and MS experiments, respectively, to improve the statistical power to be able to identify the differences between resting and depolarized conditions. Four biological replicates were chosen for PL-CLIP, three for PL-Ribo-seq, five for PL-MS and five for eIF4G2 CLIP. For all the other streptavidin immunoprecipitation and reporter experiments, at least three biological replicates were chosen. For the imaging studies, at least two biological replicates were chosen with multiple fields from each biological replicate that would represent the whole slide, and the number of images and fields studied for each figure is reported in the corresponding figure legends.

Data distribution was assumed to be normal, but this was not formally tested. The details of all the statistical tests used are reported in the respective figure legends.

The primary cortical neuron cell culture plates were randomized when deciding resting versus activated conditions or Pan-TurboID versus TurboID-PSD95 virus addition. All the resting and activated neuronal samples from the same biological replicate were processed simultaneously for all the experiments.

Processing of the reporter samples and imaging of resting versus activated neurons were blinded. Further blinding was not possible during the preparation of samples for RNA-seq, ribosome profiling, MS and CLIP because different conditions needed to be identified for downstream processing.

### Reporting summary

Further information on research design is available in the Nature Portfolio Reporting Summary linked to this article.

## Online content

Any methods, additional references, Nature Portfolio reporting summaries, source data, extended data, supplementary information, acknowledgements, peer review information; details of author contributions and competing interests; and statements of data and code availability are available at 10.1038/s41593-024-01615-5.

### Supplementary information

Reporting Summary

Supplementary Table 1–6 Combined workbook including six supplementary tables in order: Supplementary Table 1. Dendritically enriched RNAs by resting, depolarized and differential PL-CLIP. Supplementary Table 2. Dendritically enriched proteome by resting and differential PL-MS. Supplementary Table 3. Dendritically translated RNAs by resting, depolarized and differential PL-Ribo-seq. Supplementary Table 4. uORFs with and without start codon overlaps detected by ORF-RATER. Supplementary Table 5. Dendritic RNAs with increased 5′ UTR translation and increased or decreased CDS translation upon depolarization. Supplementary Table 6. RNAs that are bound more by eIF4G2 in their 5′ UTRs upon depolarization.

### Source data

Source Data Fig. 1 Unprocessed western blots for Fig. 1b.

Source Data Fig. 3 Unprocessed western blots for Fig. 3a.

Source Data Fig. 4 Unprocessed western blots for Fig. 4b,d,g.

Source Data Extended Data Fig. 1 Unprocessed western blots for Extended Data Fig. 1f,h–j.

Source Data Extended Data Fig. 5 Unprocessed western blots for Extended Data Fig. 5a.

Source Data Extended Data Fig. 6 Unprocessed western blots for Extended Data Fig. 6d,g,h.

Source Data Extended Data Fig. 8 Unprocessed western blots for Extended Data Fig. 8b,e.

Source Data Extended Data Fig. 9 Unprocessed western blots for Extended Data Fig. 9b,d,f,j.

Source Data Extended Data Fig. 10 Unprocessed western blots for Extended Data Fig. 10d.

## Data Availability

All sequencing data generated in this study were deposited in the Gene Expression Omnibus with accession number GSE213083. Proteomics data were deposited in the ProteomeXchange Consortium via the PRIDE79 partner repository with dataset identifier PXD050222. The databases used in this study include the Mouse UniProt proteome FASTA database (March 2020), the mm10 UCSC mouse genome and RBPmap version 1.1 (http://rbpmap.technion.ac.il/). We have the rights to publish BioRender figures, and Figs. 1a,d, 3a, 4a and 6 were created with BioRender. All other data used in this study are available or are described in the paper or supplementary materials. Source data are provided with this paper.

## Code availability

All packages and software described in the Methods section were used according to standard protocols. Custom code was not generated in this study.
