# Supplementary figures and images for: Neuronal activity rapidly reprograms dendritic translation via eIF4G2:uORF binding
Source: Nat Neurosci. 2024 Apr 8;27(5):822–35. doi: 10.1038/s41593-024-01615-5 (PMC11088998; doi:10.1038/s41593-024-01615-5)

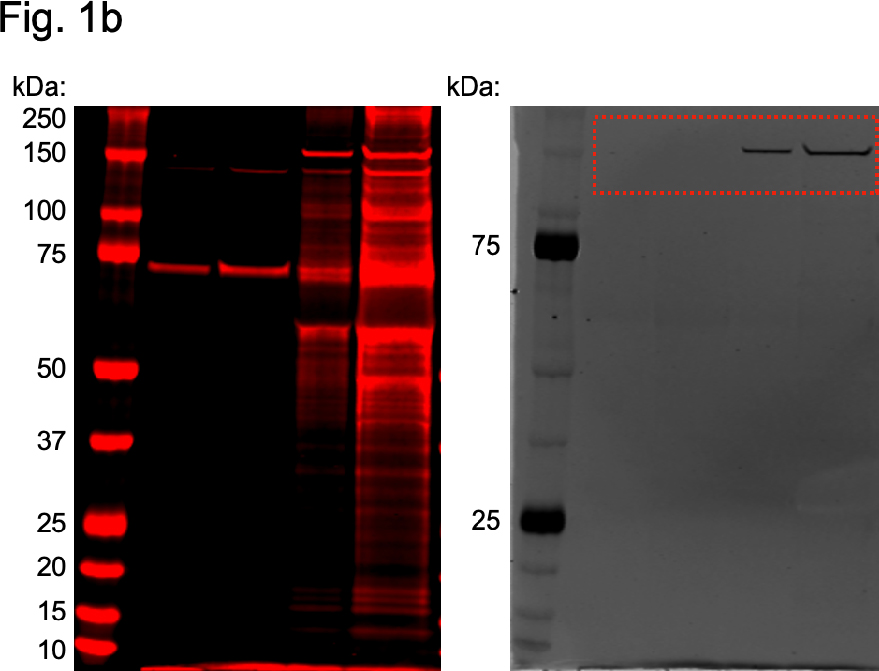

Supplement: Supplementary file 3 — Unprocessed western blots for Fig. 1b. [file 41593_2024_1615_MOESM3_ESM.jpg]

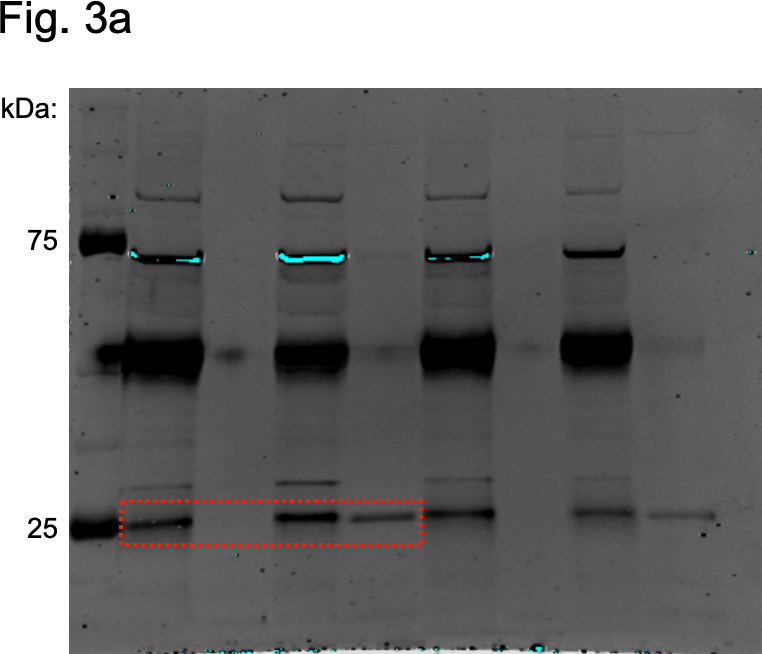

Supplement: Supplementary file 4 — Unprocessed western blots for Fig. 3a. [file 41593_2024_1615_MOESM4_ESM.jpg]

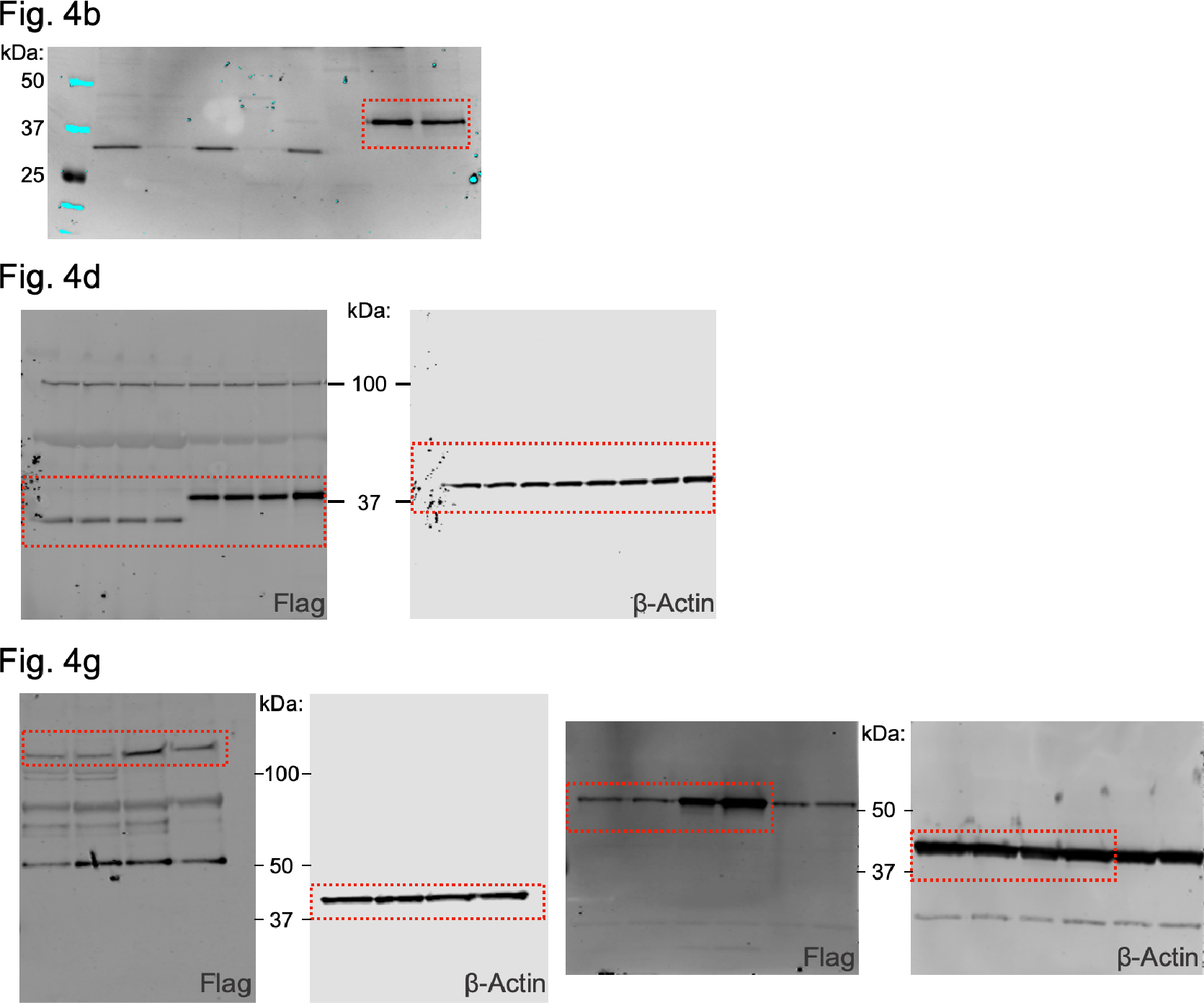

Supplement: Supplementary file 5 — Unprocessed western blots for Fig. 4b,d,g. [file 41593_2024_1615_MOESM5_ESM.jpg]

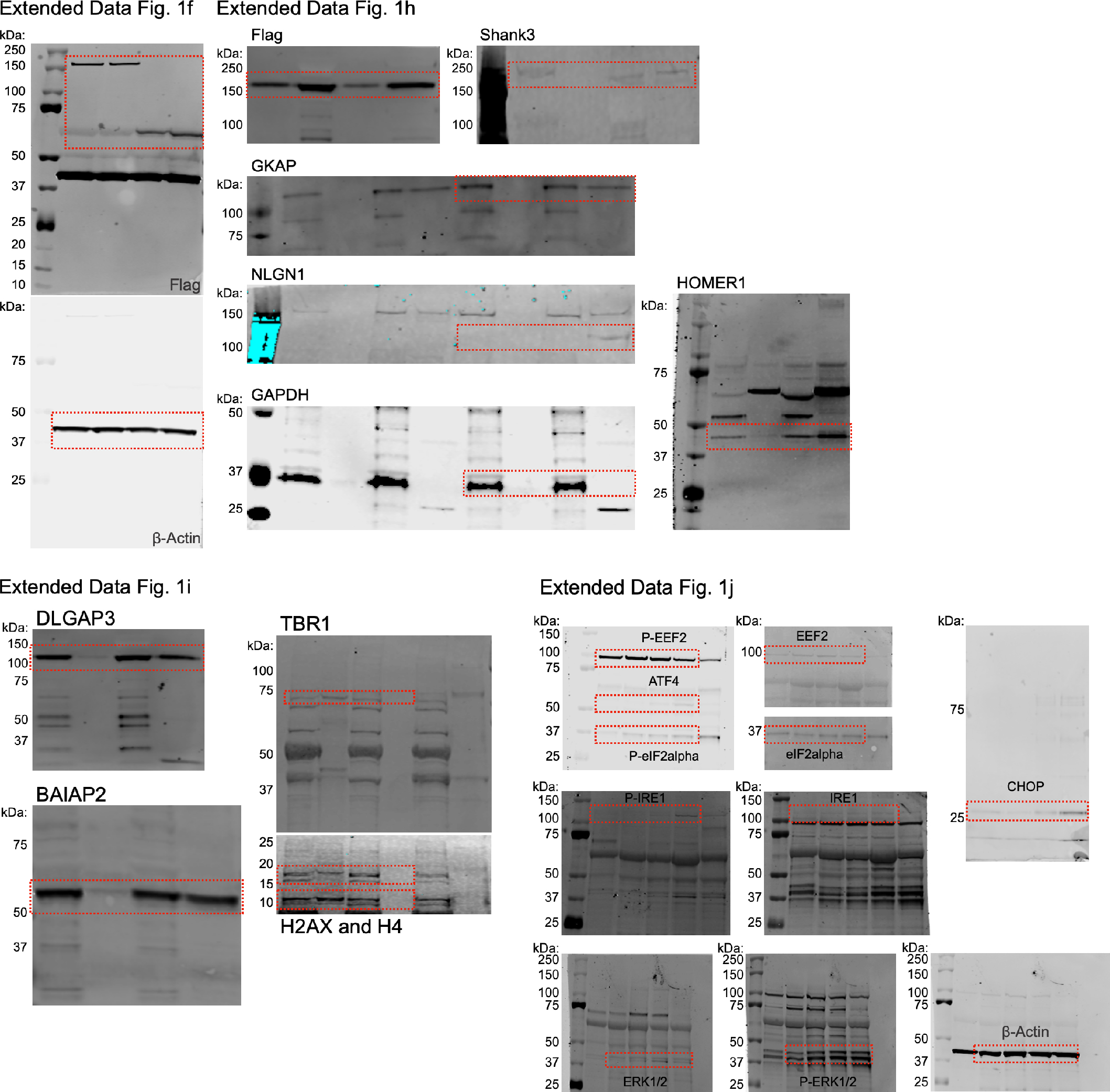

Supplement: Supplementary file 6 — Unprocessed western blots for Extended Data Fig. 1f,h–j. [file 41593_2024_1615_MOESM6_ESM.jpg]

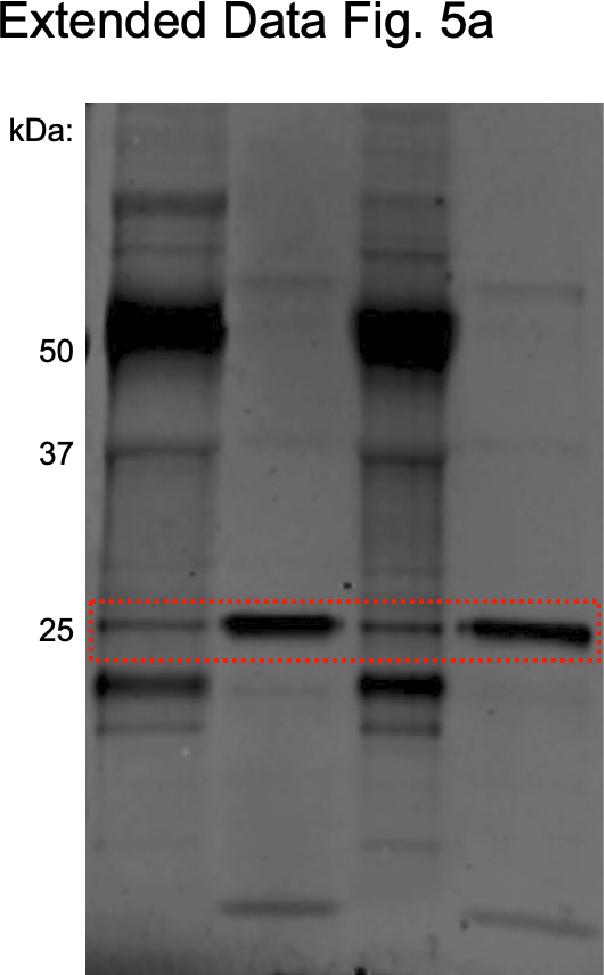

Supplement: Supplementary file 7 — Unprocessed western blots for Extended Data Fig. 5a. [file 41593_2024_1615_MOESM7_ESM.jpg]

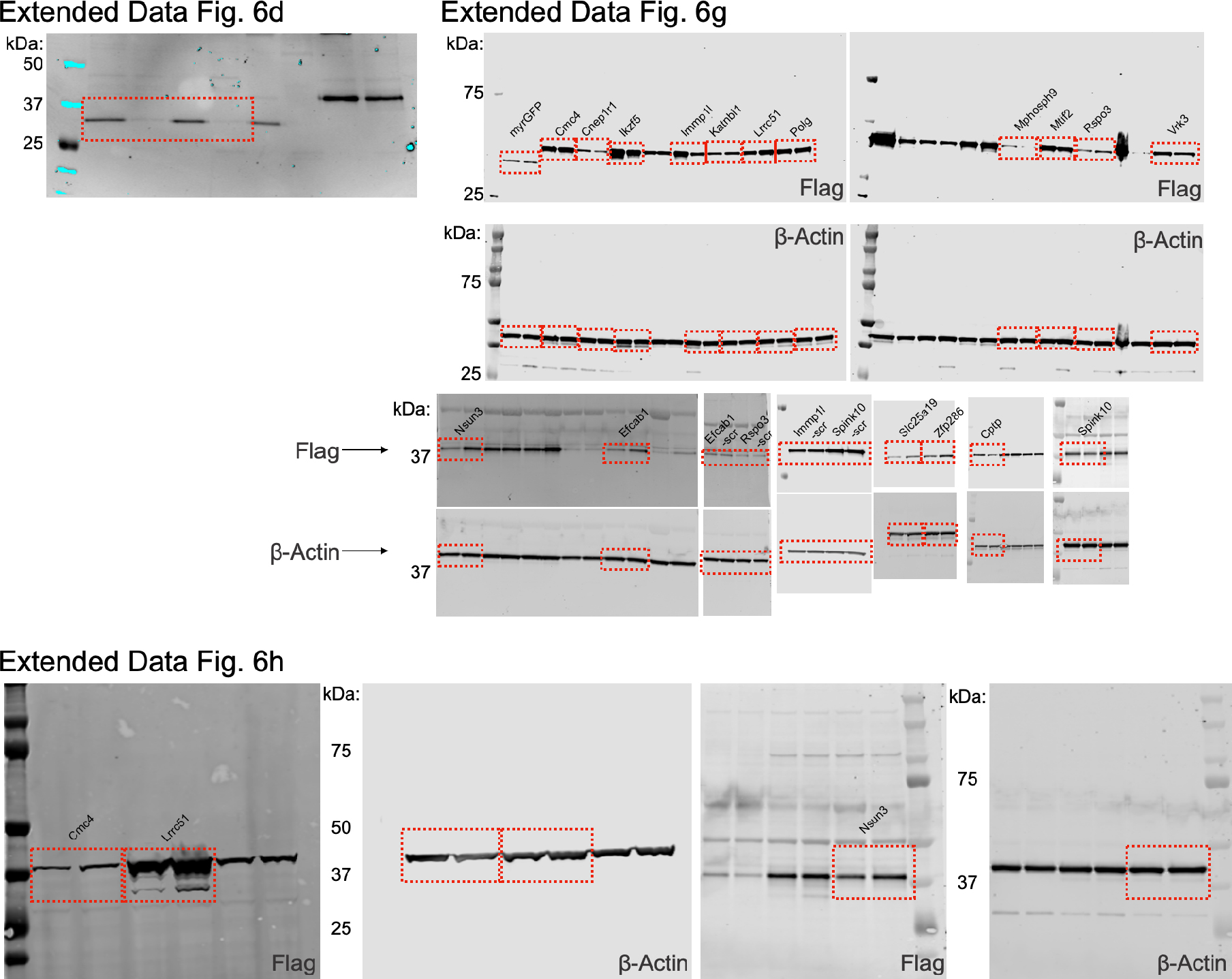

Supplement: Supplementary file 8 — Unprocessed western blots for Extended Data Fig. 6d,g,h. [file 41593_2024_1615_MOESM8_ESM.jpg]

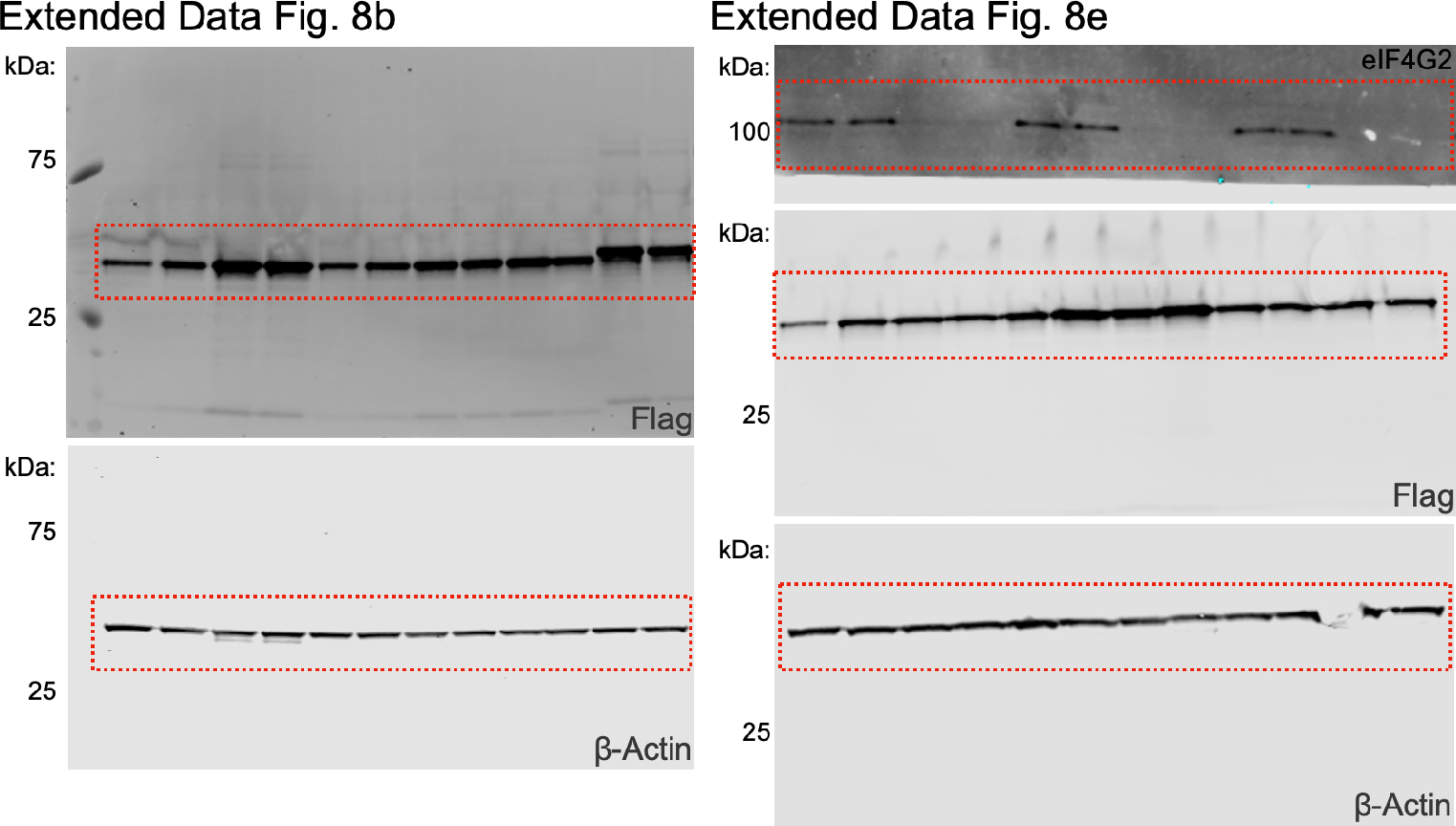

Supplement: Supplementary file 9 — Unprocessed western blots for Extended Data Fig. 8b,e. [file 41593_2024_1615_MOESM9_ESM.jpg]

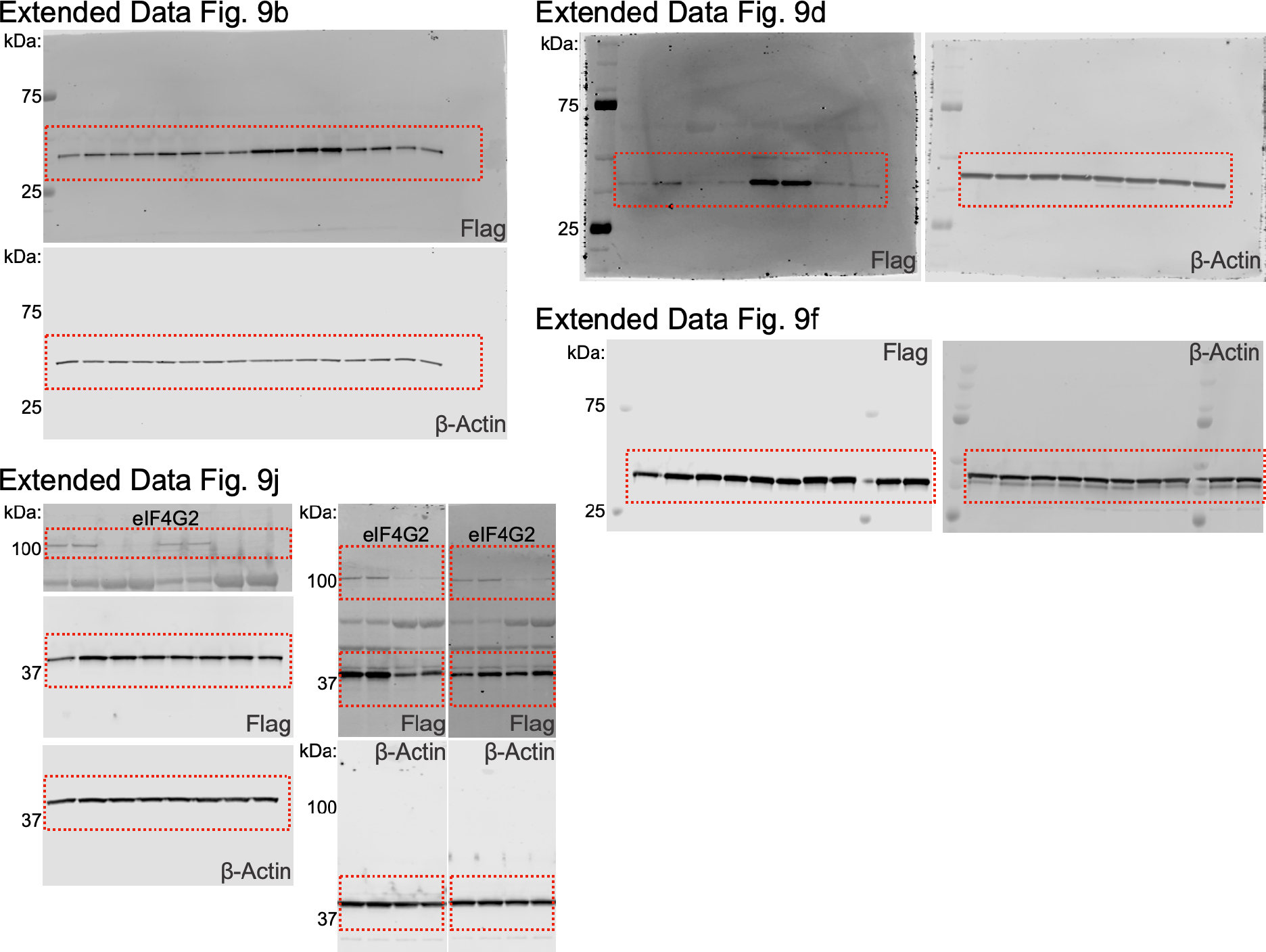

Supplement: Supplementary file 10 — Unprocessed western blots for Extended Data Fig. 9b,d,f,j. [file 41593_2024_1615_MOESM10_ESM.jpg]

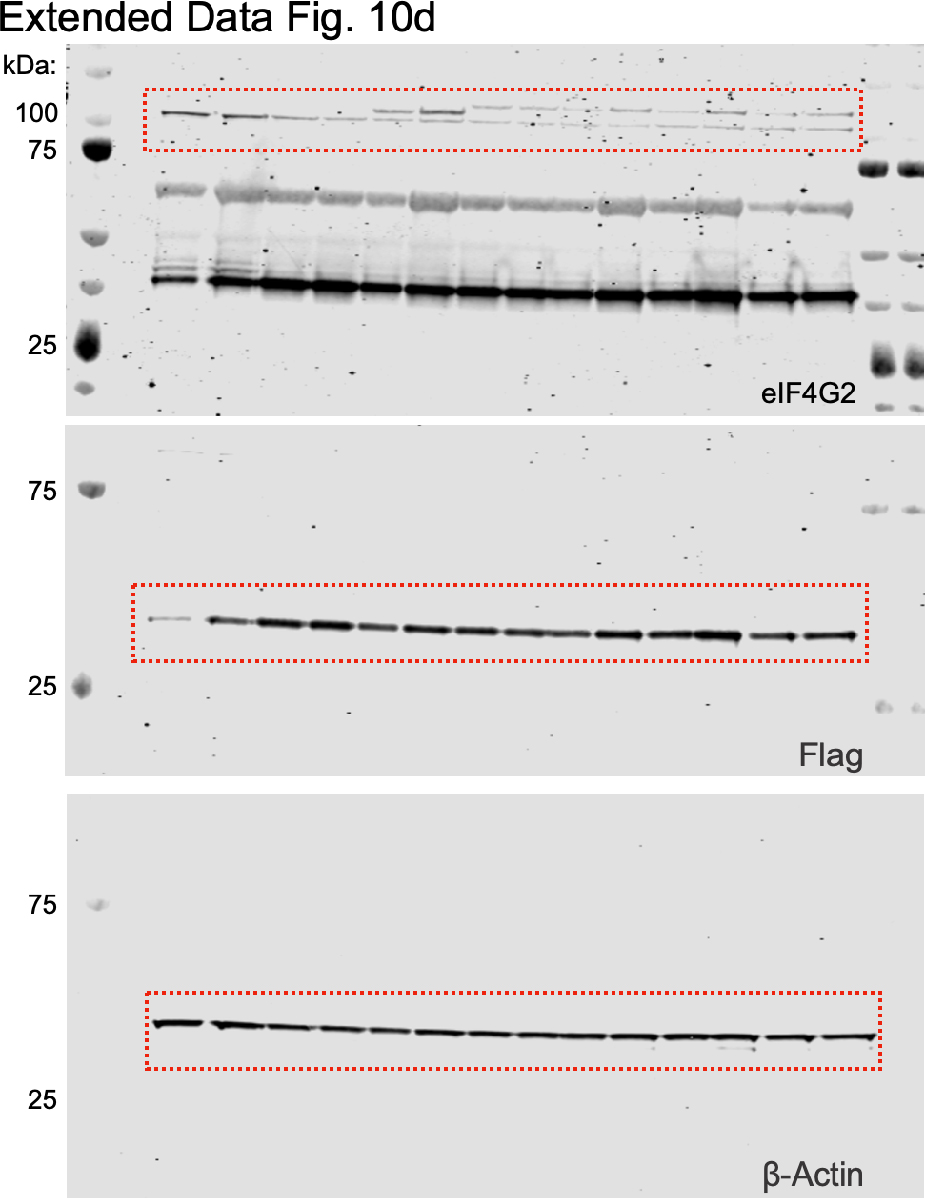

Supplement: Supplementary file 11 — Unprocessed western blots for Extended Data Fig. 10d. [file 41593_2024_1615_MOESM11_ESM.jpg]
